# Interleukin-11 Signaling in Liver Disease: Mechanisms, Cellular Crosstalk, and Therapeutic Potential

**DOI:** 10.3390/biom16091309

**Published:** 2026-09-09

**Authors:** Zhiyuan Chen, Fan Yu, Yanhua Qiu, Jun Lin, Meilian Yu, Jinwei Yan, Xianzhi Liu

**Affiliations:** 1 Department of Gastroenterology, Xiang’an Hospital of Xiamen University, School of Medicine, Xiamen University, Xiamen 361000, China; 2 Department of Gastrointestinal Surgery, Xiang’an Hospital of Xiamen University, School of Medicine, Xiamen University, Xiamen 361000, China; 3 Department of Anesthesiology, West China Hospital of Sichuan University, Chengdu 610041, China; 4 Department of Pediatrics, Xiang’an Hospital of Xiamen University, School of Medicine, Xiamen University, Xiamen 361000, China

**Keywords:** interleukin-11, liver disease, cellular crosstalk, targeted therapy

## Abstract

The liver has a remarkable regenerative capacity, but persistent injury caused by drugs, metabolic dysfunction, alcohol, and chronic inflammation can overwhelm this process and promote fibrosis and hepatocellular carcinoma. Interleukin-11 (IL-11), a cytokine that signals through glycoprotein 130 (gp130), was historically considered hepatoprotective; however, accumulating experimental evidence predominantly identifies endogenous IL-11 as an amplifier of liver injury and maladaptive repair. In hepatocytes, IL-11 promotes oxidative stress, mitochondrial dysfunction, and cell death while limiting effective regeneration. In hepatic stellate cells, it sustains myofibroblastic activation and extracellular matrix production, while crosstalk with hepatocytes, macrophages, and the matrix reinforces a fibroinflammatory niche. In hepatocellular carcinoma, experimental and associative clinical evidence implicates IL-11 in tumor growth, invasion, metastatic colonization, postoperative recurrence, stromal remodeling, and immune suppression. These findings have supported the development of therapeutic strategies targeting the IL-11/IL-11RA axis, with the potential to provide broad therapeutic benefits across a wide spectrum of liver diseases. This review summarizes IL-11 regulation and signaling in liver disease; discusses its roles in liver injury, fibrosis, and hepatocellular carcinoma; and evaluates the opportunities and challenges of IL-11-targeted therapy.

## 1. Introduction

The liver performs essential functions in metabolism, detoxification, immune tolerance, and tissue regeneration, yet it is chronically exposed to drug toxicity, lipid overload, alcohol stimulation, ischemia–reperfusion injury, gut-derived inflammatory signals, and pathogen-associated stimuli [1]. In most cases, transient liver injury can be resolved through inflammation resolution, hepatocyte proliferation, and provisional matrix remodeling [2]. However, when injury persists or the repair program becomes imbalanced, hepatocyte death, oxidative stress, inflammatory cell recruitment, hepatic stellate cell (HSC) activation, and extracellular matrix (ECM) deposition can amplify one another, gradually shifting tissue repair toward fibroinflammatory remodeling [3,4]. Thus, the progression of liver disease is not simply the consequence of single-cell damage but rather a tissue ecosystem alteration driven by sustained interactions among parenchymal cells, stromal cells, immune cells, and the matrix environment.

In this process, cytokine signaling determines whether the injury response proceeds toward effective regeneration or evolves into persistent inflammation and fibrosis [5]. Interleukin-11 (IL-11), a glycoprotein 130 (gp130)-dependent member of the IL-6 cytokine family, has emerged as an important regulatory factor in this network. Existing evidence suggests that the role of IL-11 in the liver depends on the disease context, cellular source, receptor distribution, and duration of signaling [6,7]. During acute injury, IL-11 can participate in hepatocyte stress responses and influence the extent of cell death and the quality of regeneration [8]. Under conditions of persistent injury, IL-11 may further coordinate interactions among hepatocyte damage, HSC activation, inflammatory amplification, and matrix deposition, thereby driving the transition from reversible repair to chronic fibrotic remodeling [7]. Although drug-induced liver injury, metabolic dysfunction-associated steatotic liver disease, and alcohol-associated liver disease arise from distinct initiating insults, their progressive forms share several pathological features, including hepatocellular stress and death, oxidative and mitochondrial dysfunction, inflammation, HSC activation, and fibrotic remodeling [4,9]. Within this pathological setting, IL-11 may contribute to autocrine and paracrine communication among hepatocytes, HSCs, immune cells, and other components of the hepatic microenvironment. As liver disease progresses, sustained IL-11-related signaling could potentially extend beyond tissue injury and fibrotic remodeling to influence the development of a tumor-promoting hepatic microenvironment [10,11]. In this context, IL-11 may represent a mechanistic link connecting hepatocellular injury, chronic fibrosis, and hepatocarcinogenic remodeling.

Based on this, the present review focuses on the role of IL-11 in liver diseases, with emphasis on its pathological significance in liver injury responses, fibrosis progression, intercellular communication, and hepatocellular carcinoma (HCC) remodeling, and evaluates its translational potential as a therapeutic target. By integrating existing evidence, this review aims to provide a reference for understanding the functional positioning of IL-11 in liver disease progression and for future interventional studies.

## 2. Basic Characteristics and Biological Functions of IL-11 Signaling

### 2.1. Molecular Architecture of IL-11 and Its Receptor Signaling Complex

IL-11 is a member of the IL-6 cytokine family and a typical gp130-dependent cytokine [12]. The human IL-11 gene is located on the long arm of chromosome 19 (19q13.3–13.4), spans approximately 7 kb, and consists of five exons and four introns [13]. The mature peptide contains 178 amino acid residues with a molecular weight of approximately 19 kDa and adopts a type I four-α-helix bundle conformation [14,15]. Signal transduction depends on the assembly of a receptor complex comprising the ligand-specific receptor IL-11Rα and the common signaling receptor gp130. IL-11 first binds to IL-11Rα, after which gp130 is recruited, ultimately forming a high-affinity hexameric signaling platform composed of two IL-11, two IL-11Rα, and two gp130 molecules [16,17].

### 2.2. IL-11 Within the IL-6 Cytokine Family: Expression, Receptor Biology, and Functional Divergence

#### 2.2.1. Expression Patterns and Cellular Sources

IL-11 was first identified in 1990 by Paul et al. as a soluble factor secreted by the primate bone marrow stromal cell line PU-34. It was initially characterized by its ability to stimulate the proliferation of an IL-6-dependent murine plasmacytoma cell line and to support lymphohematopoietic and megakaryocytic development [14]. Subsequent studies established its thrombopoietic activity and its capacity to increase circulating platelet counts [18]. With further investigation, however, IL-11 has been recognized as more than a hematopoietic cytokine; its functional repertoire has expanded considerably, encompassing embryonic implantation, placental development, immune microenvironment modulation, injury-associated tissue remodeling, and many other biological processes [19,20,21]. Subsequent molecular characterization showed that IL-11 adopts the long-chain four-α-helix bundle structure typical of IL-6 family cytokines and signals through a receptor complex containing the shared signal-transducing subunit gp130, thereby establishing IL-11 as a member of this family [15]. The main members of the IL-6 family currently include IL-6, IL-11, leukemia inhibitory factor (LIF), oncostatin M (OSM), and ciliary neurotrophic factor (CNTF).

Under physiological conditions, IL-11 transcription and translation are generally tightly restricted, and marked elevations are rarely detectable in the peripheral blood of healthy individuals [22,23]. In contrast, IL-11 expression is upregulated during tissue injury, chronic inflammation, fibrosis, and solid-tumor development, with reported cellular sources including stromal and mesenchymal cells, epithelial and parenchymal cells, endothelial cells, and tumor cells, whose relative contributions vary according to tissue, stimulus, disease stage, and experimental model [18,24,25,26]. This expression pattern overlaps substantially with those of other IL-6 family cytokines, whose cellular sources also vary with tissue and pathological context. Cellular origin alone therefore does not account for their functional divergence, which also depends on receptor distribution and the organization of the downstream signaling complex.

#### 2.2.2. Receptor Composition and Cellular Distribution

The major IL-6 family cytokines share gp130 as a signal-transducing receptor subunit but use different ligand-binding receptors and receptor assemblies. IL-6 and IL-11 bind IL-6Rα and IL-11Rα, respectively, before inducing gp130 homodimerization. LIF signals through a gp130–LIFRβ heterodimer. OSM uses gp130 together with OSMRβ or, in humans, LIFRβ, whereas CNTF first binds CNTFRα and then recruits gp130 and LIFRβ [12,15]. Thus, despite the widespread expression of gp130, cellular responsiveness is determined by the availability of the corresponding ligand-specific receptor and coreceptor components.

The cellular sources of IL-11 in pathological states are diverse, and its effects through conventional cis-signaling depend largely on the distribution of IL-11Rα. IL-11Rα expression has been reported in stromal and mesenchymal populations, including fibroblasts, vascular smooth muscle cells, pericytes, and hepatic stellate cells, as well as in selected epithelial and parenchymal cells, such as hepatocytes, alveolar epithelial cells, and renal tubular epithelial cells [27,28,29,30]. The abundance and functional relevance of IL-11Rα may nevertheless vary with tissue, cellular state, and disease context, and transcript expression does not necessarily indicate the presence of a signaling-competent receptor at the cell surface.

Furthermore, membrane-bound IL-11Rα can be proteolytically shed to generate a soluble ectodomain variant, sIL-11Rα. The resulting IL-11–sIL-11Rα complex can initiate trans-signaling in cells that express gp130 but lack the membrane-bound receptor, thereby broadening the spectrum of IL-11-responsive cells [31,32,33]. Several proteases have been identified as mediators of IL-11Rα shedding, including the metalloprotease ADAM10, the serine proteases neutrophil elastase, proteinase 3, and the rhomboid-like protease RHBDL2 [31,34]. This mechanism resembles the well-established trans-signaling pathway of IL-6, although the conditions under which IL-11 trans-signaling occurs in vivo and its contribution to tissue-specific responses remain incompletely understood.

#### 2.2.3. Shared Signaling Pathways and Functional Divergence

IL-6 family cytokines activate a common intracellular signaling repertoire centered on JAK–STAT, SHP2–RAS–ERK, and PI3K–AKT pathways. For IL-6 and IL-11, the ligand-binding α-receptors lack intrinsic kinase activity, and signal transduction is initiated by gp130-associated JAKs. Phosphorylated gp130 provides docking sites for STAT1 and STAT3, as well as SHP2-containing adaptor complexes that connect receptor activation to ERK and other downstream pathways [12,15]. This common signaling scaffold accounts for the substantial overlap among family members in regulating survival, proliferation, differentiation, inflammation, and tissue repair.

Receptor architecture influences how this shared repertoire is used. IL-6 and IL-11 both signal through gp130 homodimers, making the distribution of IL-6Rα and IL-11Rα, ligand–receptor affinity, receptor trafficking, and signaling duration important determinants of their functional divergence. LIF, OSM, and CNTF recruit LIFRβ or OSMRβ together with gp130, introducing additional intracellular docking motifs that can alter the relative activation of STAT1, STAT3, ERK, and AKT. Structural studies further indicate that productive signaling depends on more than ligand binding and initial complex assembly: changes in gp130 orientation, complex stability, or conformational dynamics can alter downstream responses [15,17,35].

Signal amplitude and duration add another level of specificity to gp130-dependent signaling. Together with receptor abundance, SOCS3-dependent negative feedback, SHP2 recruitment, and concurrent inflammatory, metabolic, or mechanical signals, these factors influence whether receptor activation supports acute adaptation, survival, regeneration, inflammation, or longer-term tissue remodeling [12,15,36,37]. Within this framework, IL-6 is closely associated with immune regulation and acute-phase responses; LIF and CNTF have prominent developmental, reparative, and neurotrophic functions; and OSM frequently coordinates leukocyte–stromal communication during inflammation and remodeling [12,15,37]. IL-11 participates in hematopoietic support, reproduction, immune regulation, and injury-associated tissue responses [18,38]. In the liver, the consequences of gp130 activation therefore depend on the responsive cell population, receptor configuration, and signaling kinetics, providing a basis for the divergent effects of individual IL-6 family cytokines.

### 2.3. Roles of IL-11 in Liver Diseases

#### 2.3.1. Early Pharmacological Studies and the Hepatoprotective Interpretation

The early use of recombinant human IL-11 (rhIL-11) in rodent liver models reflected its pharmacological development. rhIL-11 was the first IL-11 preparation produced at pharmaceutical scale and developed clinically for chemotherapy-induced thrombocytopenia [14,18]. It was biologically active in rodents, dogs, and nonhuman primates and showed specific activity similar to that of recombinant mouse IL-11 (rmIL-11) in a murine hematopoietic proliferation assay [39], suggesting functional conservation across species. These observations made rhIL-11 a practical and seemingly appropriate reagent for studying IL-11 activity in rodent models. Early liver studies therefore focused on the pharmacological effects of rhIL-11 without separately defining the function of endogenous mouse Il11.

Against this background, rhIL-11 was tested in rodent models of liver injury induced by concanavalin A, acetaminophen (APAP), endotoxin, D-galactosamine, and ischemia–reperfusion. Its administration reduced inflammatory mediators, biochemical and histological injury, or mortality, leading to the early view of IL-11 as an anti-inflammatory and hepatoprotective cytokine [40,41,42,43,44]. Endogenous IL-11 was subsequently linked to STAT3 activation and compensatory hepatocyte proliferation after oxidative liver injury, further supporting a possible role in tissue repair [45].

Notably, the early evidence for a beneficial effect of pharmacological IL-11 was not confined to rodent models. In a small open-label pilot study, Lawitz et al. administered rhIL-11 to 20 patients with chronic hepatitis C virus (HCV) infection, advanced fibrosis, and previous failure of antiviral therapy. Twelve weeks of rhIL-11 treatment reduced histological inflammatory activity and serum alanine aminotransferase (ALT), although fibrosis stage did not significantly improve and HCV RNA increased during treatment [46]. These findings suggested a short-term anti-inflammatory effect of exogenous rhIL-11 in chronic HCV infection but did not establish a direct hepatoprotective, antifibrotic, or disease-modifying role for endogenous IL-11.

#### 2.3.2. Species Dependence and Reinterpretation of IL-11 Activity

The early hepatoprotective interpretation began to change in 2017, when Schafer et al. found that rhIL-11 activated human fibroblasts but showed little profibrotic activity in mouse fibroblasts, while rmIL-11 strongly activated mouse cells [24]. This observation prompted subsequent liver studies to use species-matched ligands together with genetic and antibody-based approaches. In mouse models of steatohepatitis and APAP injury, these complementary approaches identified IL-11 as an amplifier of hepatocyte death and fibrosis and as a constraint on regeneration [8,25]. This conclusion directly opposed the hepatoprotective interpretation derived from rhIL-11 administration in rodents.

**Figure 1:**
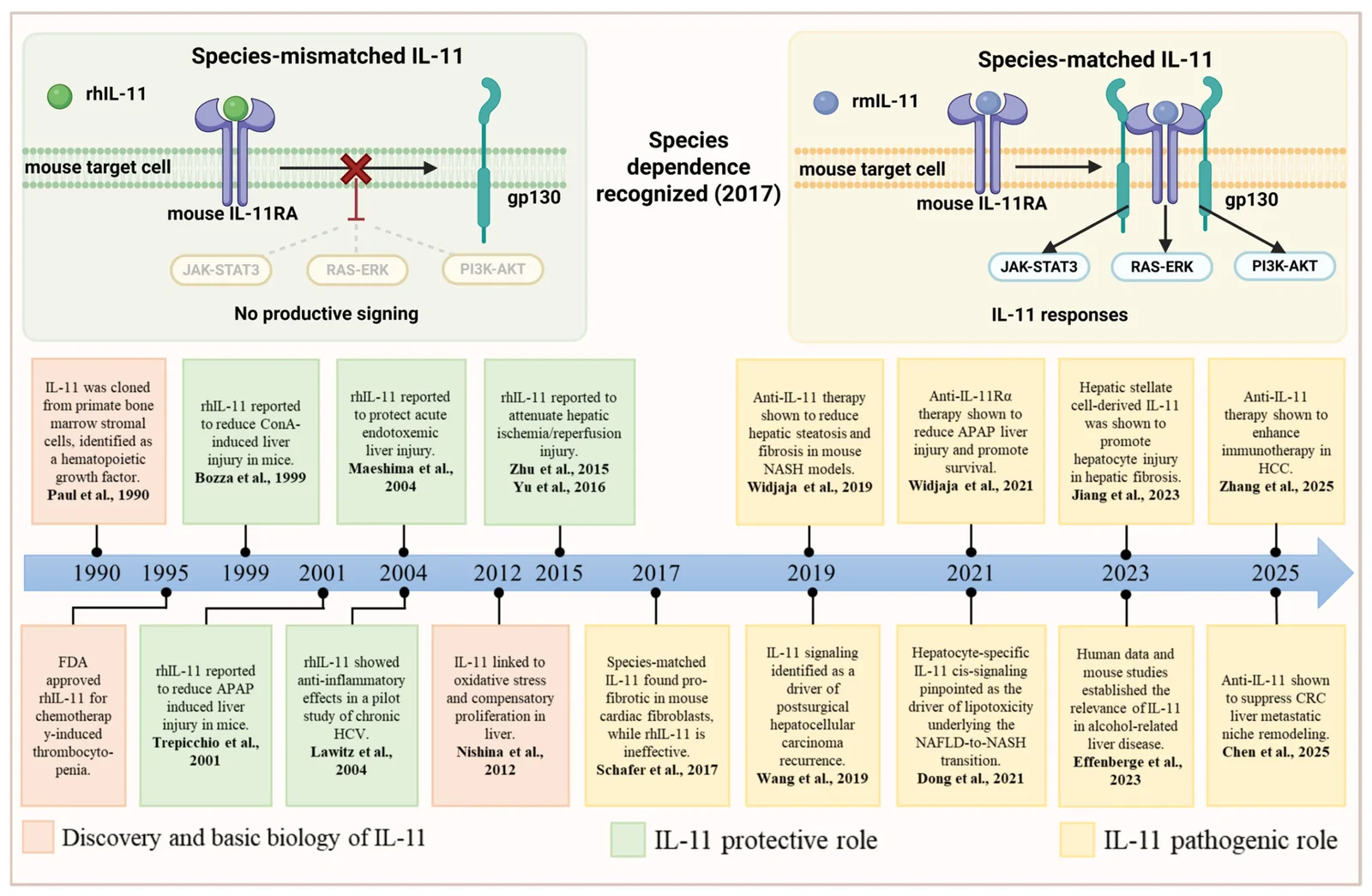
Historical evolution of IL-11 research in liver disease. Refs. [8,14,24,25,28,40,41,42,43,44,45,46,47,48,49,50,51].

To resolve this apparent contradiction, researchers directly compared species-matched and species-mismatched IL-11. In mouse hepatocytes, rmIL-11 induced sustained ERK/JNK activation and cell death, whereas rhIL-11 failed to reproduce these responses; the reciprocal ligand dependence was observed in human hepatocytes [8]. rhIL-11 nevertheless retained affinity for mouse IL-11RA and competed with rmIL-11 for receptor binding, thereby inhibiting rmIL-11-induced signaling and cytotoxicity. Although high concentrations of rhIL-11 could produce transient STAT3 phosphorylation, they did not elicit the sustained ERK/JNK signaling characteristic of species-matched IL-11 [8]. Thus, rhIL-11 can functionally antagonize endogenous mouse IL-11 rather than act as an equivalent agonist, explaining why its administration appeared hepatoprotective in earlier rodent studies (Figure 1). To facilitate comparison of these apparently divergent findings, the experimental and clinical evidence supporting protective, indirectly protective, pathogenic, or model-specific roles of IL-11 across liver diseases is summarized in Table 1.

This uncoupling of receptor binding from productive signaling may arise during higher-order assembly of the IL-11–IL-11RA–gp130 complex. IL-11 initially binds IL-11RA through its site I interface, after which additional ligand surfaces engage gp130 and promote formation of a signaling-competent hexameric complex [16,17]. Species-specific sequence differences could preserve the initial rhIL-11–mouse IL-11RA interaction while impairing subsequent gp130 recruitment, receptor orientation, or cooperative complex assembly. The resulting complex may be incomplete, geometrically distorted, or insufficiently stable to sustain downstream signaling. Such partial assembly could permit brief STAT3 activation at high ligand concentrations while failing to generate the signal strength or duration required for sustained ERK/JNK activation. Differences in complex residence time, assembly kinetics, or receptor trafficking may also contribute, although these possibilities remain untested. Engineered IL-11 muteins and structural studies of gp130 variants support the broader principle that alterations affecting later assembly steps can uncouple receptor occupancy from signaling output [17,35]. Because the rhIL-11–mouse IL-11RA–gp130 complex has not been structurally resolved, the precise molecular basis remains uncertain. These findings shifted the field from the pharmacological effects of exogenous rhIL-11 toward the functions of endogenous, species-matched IL-11 in liver disease. However, species mismatch does not account for all earlier protective observations, as the HCV pilot study used rhIL-11 in human subjects [46]. The reduction in hepatic inflammation and ALT, together with increased HCV RNA, may reflect context-dependent immunomodulation rather than direct hepatoprotection. Whether pharmacological IL-11 can exert beneficial effects in selected human inflammatory settings therefore remains unresolved.

#### 2.3.3. Emerging Importance of IL-11 Within the IL-6 Cytokine Family in Liver Disease

Within the liver, IL-6 is most strongly associated with acute-phase responses and hepatocyte regeneration, whereas LIF and OSM participate in context-dependent inflammatory, reparative, and remodeling responses [11]. IL-11 is induced in response to diverse forms of hepatic stress. Lipotoxicity, oxidative stress, inflammatory and profibrotic mediators, and tissue remodeling can increase IL-11 production in different hepatic compartments [8,24,25]. Through local autocrine and paracrine signaling, IL-11 can connect parenchymal dysfunction and impaired regeneration with stromal activation and changes in the inflammatory and vascular microenvironment. These processes contribute in different proportions to acute toxic injury, metabolic and inflammatory liver disease, fibrosis, cirrhosis, and liver cancer. The convergence of multiple upstream stimuli and multicellular responses may therefore explain the apparent prominence of IL-11 across the spectrum of liver disease.

## 3. IL-11 Production and Downstream Signal Transduction

### 3.1. Regulation of IL-11 Production

#### 3.1.1. Transcriptional Regulation

The IL11 promoter integrates diverse upstream signals arising from inflammation, oxidative stress, hypoxia, and matrix remodeling. Regulatory axes involving JAK/STAT3, ROS/ERK–Fra-1, HIF-1/MAFF–BACH1, TGF-β/SMAD–Runx2, and Ras/MAPK–AP-1 can promote IL11 transcription or enhance IL-11 expression in a cell context-dependent manner. Inflammatory stimuli can activate JAK/STAT3 signaling, leading to STAT3 tyrosine phosphorylation, homodimerization, and nuclear translocation. Activated STAT3 can then directly bind to STAT3-responsive regions within the IL11 promoter and enhance its transcription [56,68]. The MCM7–SHCBP1–RACGAP1 axis can further promote STAT3 phosphorylation and nuclear translocation during this process, thereby strengthening STAT3-dependent IL11 promoter activation [56]. Oxidative stress can induce IL11 transcription through the ROS–ERK–Fra-1 axis. ERK-dependent phosphorylation stabilizes Fra-1 and promotes its binding to AP-1-responsive elements within the IL11 promoter [45]. NRF2-related signaling can further enhance this process by increasing Fra-1 abundance, particularly under electrophilic stress conditions [69]. Under hypoxic conditions, HIF-1 can directly or indirectly increase IL11 expression. HIF-1 can induce MAFF expression, after which MAFF can form a transcriptionally active complex with BACH1. The MAFF–BACH1 complex can bind to the MARE/ARE-containing region of the IL11 promoter and enhance its transcription [70,71]. During tissue remodeling, increased ECM deposition and crosslinking increase tissue stiffness and generate mechanical stress. Mechanical stimulation can promote IL11 transcription through ΔFosB/JunD-dependent AP-1 activity and PKCδ-mediated Smad1/5 activation [72,73]. TGF-β can independently activate the IL11 promoter through AP-1-responsive elements [74,75]. A previous study further demonstrated that TGF-β-induced Runx2 can cooperate with phosphorylated SMADs or c-Jun to enhance IL11 promoter activity [76]. These pathways can converge at the transcriptional level to reinforce IL-11 expression during tissue remodeling. Ras/MAPK signaling may further amplify IL-11 expression through AP-1-dependent transcriptional programs [77]. Collectively, IL-11 functions as a stress-responsive mediator whose transcription can be induced by cellular stress, inflammatory stimulation, and tissue remodeling.

#### 3.1.2. Post-Transcriptional Regulation

The stability and post-transcriptional availability of IL11 mRNA further determine the magnitude and persistence of IL-11 production. Reported mechanisms of post-transcriptional regulation primarily involve noncoding RNAs (ncRNAs) and N^6^-methyladenosine (m^6^A)-dependent RNA regulation. Among ncRNAs, lncRNA-ATB can directly interact with IL11 mRNA and increase its stability, thereby enhancing autocrine IL-11 production [58]. The m^6^A-modified lncRNA AC026356.1 can bind to IGF2BP1 and promote the association of IGF2BP1 with IL11 mRNA, leading to prolonged transcript stability and increased IL-11 secretion [59]. In contrast, miR-204, miR-211, and miR-379 can directly bind to the IL11 3′UTR and suppress TGF-β-induced IL-11 production [78]. miR-23b can also directly target IL11 and reduce IL-11 expression [60]. Similarly, miR-124a can directly target the IL11 3′UTR, thereby inhibiting hepatoma-cell proliferation and migration and promoting apoptosis [61]. m^6^A-dependent regulation can direct IL11 mRNA toward either degradation or stabilization, depending on the reader protein and cellular context. In HCC, YTHDF2 can recognize m^6^A-containing IL11 mRNA and promote its degradation, whereas hypoxia-induced suppression of YTHDF2 increases IL11 mRNA stability and contributes to inflammatory and vascular abnormalities [79]. By contrast, IGF2BP1 can stabilize IL11 mRNA through at least two related mechanisms. In HCC, m^6^A-modified AC026356.1 recruits IGF2BP1 and enhances its binding to IL11 mRNA [59]. A more recent study in lung adenocarcinoma further reported that IGF2BP1 can directly recognize and stabilize m^6^A-modified IL11 mRNA, thereby increasing IL-11 expression [80].

**Figure 2:**
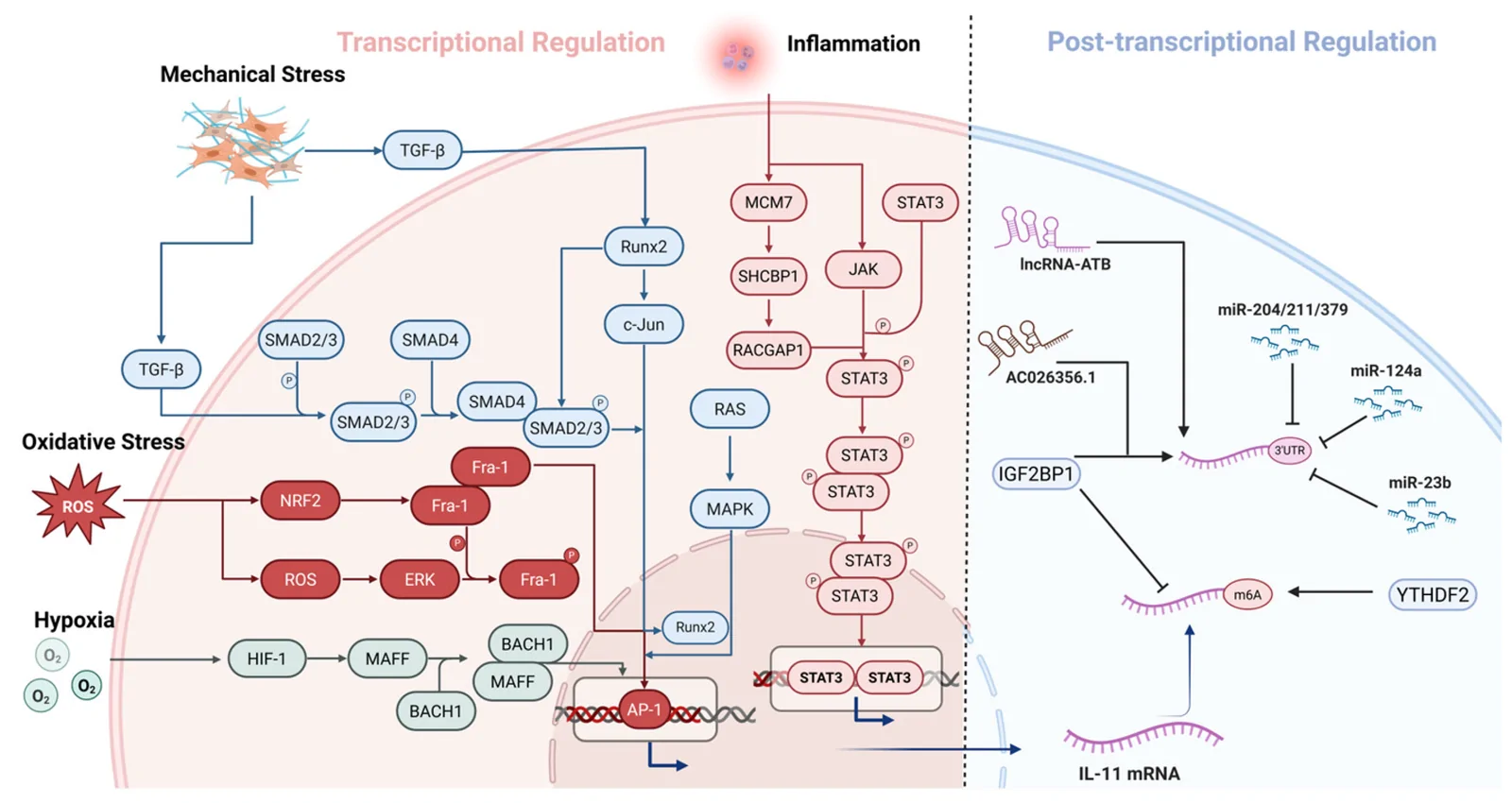
Integrated regulation of IL-11 expression.

Collectively, pathological elevation of IL-11 is determined not only by transcriptional activation but also by whether IL11 mRNA is stabilized, targeted for degradation, or maintained in a form available for protein production and secretion (Figure 2).

### 3.2. Downstream Signal Transduction

**Figure 3:**
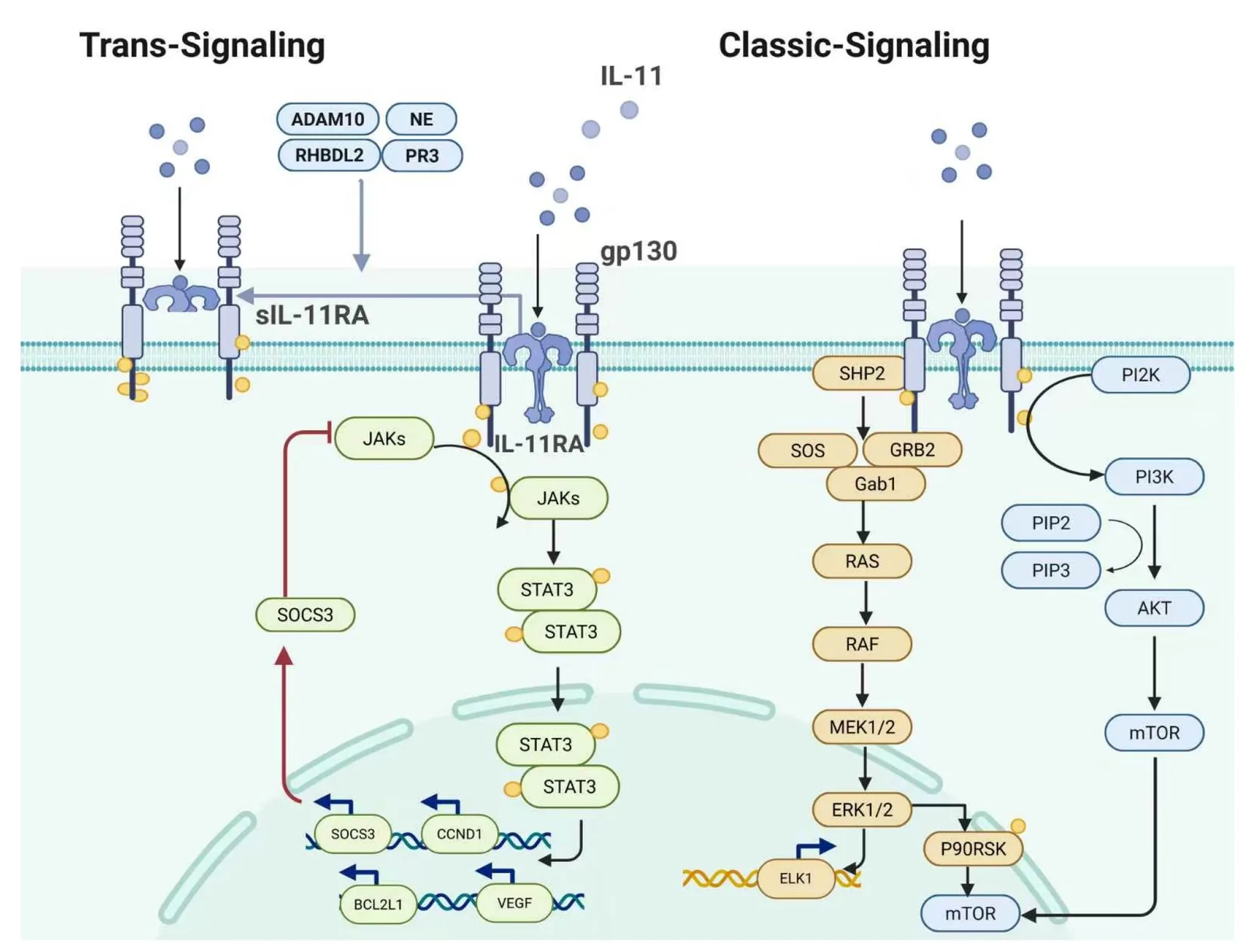
Downstream signal transduction of IL-11.

IL-11 signaling is initiated by assembly of a receptor complex comprising IL-11, IL-11Rα, and gp130. IL-11 first binds to IL-11Rα, which subsequently promotes gp130 recruitment and receptor complex formation [16]. This process induces tyrosine phosphorylation within the intracellular domain of gp130 and enables the recruitment of signaling molecules such as STAT3, SHP2, and PI3K-associated adaptors. Early studies primarily characterized IL-11 as a canonical gp130–JAK–STAT3 cytokine. More recent evidence, however, indicates that the pathological effects of IL-11 are not determined solely by STAT3-dependent transcriptional responses. RAS–ERK and PI3K–AKT–mTORC1 signaling also contribute substantially to chronic tissue injury, fibrosis, and matrix remodeling (Figure 3).

#### 3.2.1. JAK-STAT3 Signaling

The JAK-STAT3 axis represents the most extensively characterized transcriptional pathway downstream of IL-11. Following receptor complex formation, JAK family kinases associated with the cytoplasmic domain of gp130 become activated and phosphorylate specific tyrosine residues within gp130. STAT3 is subsequently recruited to these phosphotyrosine sites and phosphorylated, after which it forms dimers and translocates into the nucleus [81]. Activated STAT3 can induce the expression of genes involved in negative feedback regulation, cell-cycle progression, survival, angiogenesis, inflammation, and chemotaxis, including SOCS3, CCND1, BCL2L1, and VEGF, as well as multiple cytokine- and chemokine-related genes [81,82]. SOCS3 negatively regulates the gp130–JAK complex and limits continued signaling, whereas PIAS3 binds activated STAT3 and suppresses its DNA-binding and transcriptional activity [83,84].

#### 3.2.2. RAS-ERK Signaling

Phosphorylated gp130 can also recruit SHP2 and promote RAS activation through adaptor proteins such as Gab1, GRB2, and SOS, thereby activating the RAS–RAF–MEK1/2–ERK1/2 cascade [85]. Activated ERK can translocate into the nucleus, where it phosphorylates transcription factors such as ELK1 and regulates the expression and stability of immediate-early gene products, including c-Fos and Fra-1 [86,87]. It can also act on cytoplasmic substrates involved in protein synthesis, cytoskeletal organization, and cell motility [88,89]. Sustained IL-11-dependent ERK activation has been observed in fibroblasts and other IL-11-responsive cells and is associated with myofibroblast activation, migration, ECM production, changes in cellular morphology, and tissue remodeling [24,90]. IL-11-stimulated ERK can also activate p90RSK, which phosphorylates and inhibits LKB1, suppresses AMPK activity, and activates mTORC1 and its downstream translational machinery [91]. The ERK–p90RSK–LKB1/AMPK–mTORC1 axis therefore provides a mechanistic link between IL-11 signaling, metabolic dysfunction, increased protein synthesis, and mesenchymal remodeling.

#### 3.2.3. PI3K-AKT-mTORC1 Signaling

PI3K-AKT signaling is another established downstream branch of IL-11. Through gp130-associated adaptor complexes, IL-11 can activate PI3K, promote PIP3 formation, and recruit AKT to the plasma membrane. Activated AKT can enhance cell survival, proliferation, migration, and metabolic adaptation through downstream targets such as BAD, FOXO, GSK3β, TSC1/2, and PRAS40 [92]. AKT-mediated activation of mTORC1 further stimulates p70S6K, S6RP, and 4E-BP1-dependent translational programs [93]. IL-11-induced PI3K-AKT-mTORC1 signaling has been reported in epithelial, stromal, immune, and tumor cells and has been linked to survival, invasion, macrophage polarization, treatment resistance, and profibrotic mediator production [94,95,96,97,98]. Zhang et al. recently showed that IL-11 can promote TGF-β protein synthesis in macrophages through the PI3K-mTOR-p70S6K-S6RP pathway, thereby enhancing HSC activation and accelerating liver fibrosis progression [57]. Additional IL-11-responsive pathways have been identified in specific cell types and pathological settings. In hepatocytes, IL-11 can induce NOX4-derived ROS production, ERK and JNK activation, mitochondrial dysfunction, and cell death [28]. In renal tubular epithelial cells, IL-11 activates an ERK-p90RSK-GSK3β-SNAI1 program that promotes partial epithelial–mesenchymal transition and impairs regenerative responses [99]. In HSCs, IL-11 can activate gp130-SFK-YAP signaling, thereby promoting cell activation, contraction, migration, and collagen production [57].

Collectively, IL-11 signaling constitutes a gp130-centered network in which multiple downstream pathways operate in parallel or interact with one another. The relative contribution of each pathway depends on receptor abundance, cell type, signaling duration, and the surrounding pathological microenvironment.

## 4. IL-11-Mediated Liver Injury Responses and the Shift Toward Maladaptive Repair

### 4.1. Hepatocyte-Intrinsic IL-11 Signaling in Acute Toxic Liver Injury

The biological significance of IL-11 in the injured liver has undergone substantial reinterpretation. IL-11 was initially considered hepatoprotective because administration of rhIL-11 reduced injury in several rodent models. However, this species-mismatched ligand was subsequently shown to incompletely activate mouse IL-11 signaling and to inhibit the hepatotoxic effects of endogenous mouse IL-11 [8]. A regenerative role was also suggested by the observation that oxidative stress induced IL-11 production in damaged hepatocytes, followed by STAT3 activation and compensatory proliferation in adjacent surviving hepatocytes [45]. These findings linked IL-11 to STAT3 activation and hepatocyte cell-cycle entry but did not establish that endogenous IL-11 was required for completed cell division or restoration of functional liver mass.

Studies using species-matched ligands and loss-of-function approaches instead identified hepatocyte-derived IL-11 as a major amplifier of acute liver injury. This function has been most clearly demonstrated in acetaminophen-induced liver injury. Following acetaminophen exposure, mitochondrial dysfunction induces IL-11 secretion from damaged hepatocytes, establishing an IL-11Rα/gp130-dependent autocrine loop. This loop enhances NOX4-derived reactive oxygen species production, activates ERK and JNK, and promotes further mitochondrial dysfunction and hepatocyte death [8]. Hepatocyte-specific deletion of Il11ra1, germline deletion of Il11, and treatment with a neutralizing IL-11RA antibody all reduced APAP-induced liver injury. Importantly, suppression of IL-11 signaling is also accompanied by increased expression of cyclins and proliferating cell nuclear antigen (PCNA) and enhanced RB phosphorylation, indicating that endogenous IL-11 simultaneously amplifies injury and restrains cell-cycle re-entry during recovery [8]. Using adult mice with hepatocyte-specific deletion of either Il11 or gp130, Dong et al. further established the cellular origin and receptor dependence of this response [53]. These experiments identify stressed hepatocytes as an important source of pathological IL-11 and demonstrate that its effects require hepatocyte-intrinsic gp130 signaling, rather than merely reflecting a compensatory response to tissue damage.

Studies of ligand-specific gp130 signaling further separated the hepatotoxic effects of IL-11 from the protective effects mediated through the same coreceptor. HyperIL-6 was previously considered to enhance liver regeneration through strong STAT3 activation. However, its protective effect in acetaminophen injury was subsequently shown to arise predominantly from competition with IL-11 for gp130 engagement. HyperIL-6 reduced IL-11-associated ERK activation and hepatotoxicity independently of STAT3, whereas it conferred no additional regenerative benefit in Il11-deficient mice, which already exhibited spontaneous repair [100]. Thus, STAT3 activation and the appearance of proliferating hepatocytes do not by themselves demonstrate that IL-11 drives regeneration. They may instead reflect the attempt of surviving hepatocytes to replace cells lost through ongoing IL-11-dependent injury.

Taken together, current evidence indicates that the direct hepatotoxic effect of IL-11 outweighs its potential contribution to compensatory proliferation in APAP-induced liver injury. A context-dependent protective component nevertheless cannot be completely excluded. The reduced proliferation and prolonged injury observed in female *Il11ra1*-deficient mice [45] suggest that IL-11R signaling may contribute to recovery under particular conditions. Consistent with this possibility, an independent study found that n-3 polyunsaturated fatty acids suppressed ERK1/2–Fra-1-dependent IL-11 production, reduced downstream STAT3 phosphorylation and Bcl-2 expression, and aggravated APAP-induced injury [52]. However, the sex-dependent receptor-deficiency phenotype was not reproduced in males, while n-3 polyunsaturated fatty acids affect multiple pathways and were not accompanied by IL-11-specific rescue. Whether a beneficial signaling window exists remains unresolved, including whether it depends on the timing and duration of IL-11 exposure, injury severity, or the relative activation of STAT3 and ERK/JNK. Overall, endogenous IL-11 predominantly amplifies parenchymal loss, restricts effective regeneration, and shifts the response to acute toxic injury toward maladaptive repair.

### 4.2. IL-11 as an Amplifier of Metabolic and Alcohol-Related Liver Injury

In metabolic liver injury, IL-11 contributes to the progression from steatosis to steatohepatitis primarily by amplifying hepatocyte lipotoxic stress. Dong et al. showed that lipid loading induces an autocrine IL-11 loop in hepatocytes, leading to increased NOX4-derived ROS production, activation of ERK, JNK, and caspase-3, impaired mitochondrial function, and reduced fatty acid oxidation. These changes promote intracellular lipid accumulation, failure of metabolic adaptation, and hepatocyte death. Hepatocyte-specific deletion of Il11ra1 attenuated diet-induced steatosis, inflammation, and fibrosis and improved metabolic parameters, including circulating glucose, cholesterol, and triglyceride levels [28]. Consistently, Widjaja et al. showed that inhibition of IL-11 signaling reduced hepatocyte death, inflammation, steatosis, and fibrosis in mouse models of steatohepatitis [25]. However, the contribution of IL-11 to metabolic dysfunction-associated steatohepatitis (MASH) appears to depend on the experimental model. In a rapidly fibrosing choline-deficient, L-amino acid-defined, high-fat diet (CDAHFD) model, Il11 expression was elevated, but loss of IL-11 signaling produced only limited changes in liver injury, inflammation, fibrosis, and associated signaling pathways [54]. One possible explanation is that CDAHFD reduced hepatic ERK phosphorylation, whereas the high-fat methionine- and choline-deficient and Western diet with fructose models were associated with ERK activation. IL-11 may therefore exert its strongest effects when ERK-dependent lipotoxic stress is a prominent feature of the disease, while alternative inflammatory or fibrogenic pathways may dominate in other settings. These findings suggest that the pathological relevance of IL-11 varies with disease etiology, dietary model, and the predominant mechanism of fibrogenesis.

Evidence from Alcohol-Related Liver Disease (ALD**)** further supports a role for IL-11 as an amplifier of liver injury across distinct etiologies. Circulating and hepatic IL-11 levels increased with disease severity and were particularly elevated in patients with alcohol-associated hepatitis, while higher circulating IL-11 independently predicted poorer transplant-free survival in patients with cirrhosis [55]. In primary human hepatocytes and ethanol-fed mice, ethanol exposure enhanced IL-11 signaling together with ERK, JNK, and NOX4-related stress responses. Neutralization of IL-11RA reduced ethanol-induced liver injury, steatosis, neutrophil infiltration, and proinflammatory cytokine expression. The agreement among clinical observations, primary human hepatocytes, and mouse intervention experiments extends the injury-amplifying mechanism of IL-11 beyond dietary models, although direct therapeutic evidence remains preclinical. More recently, Li et al. reported that FP-ELNs alleviated experimental ALD, reduced circulating and hepatic IL-11 levels, and activated the PI3K/AKT/mTOR pathway, suggesting that the hepatoprotective effects of FP-ELNs are associated with suppression of IL-11-related pathological responses [101].

Collectively, these studies indicate that persistent IL-11 signaling can amplify both lipotoxic and alcohol-induced hepatocyte stress by sustaining oxidative injury, stress-kinase activation, and metabolic dysfunction. Its contribution is nevertheless shaped by the initiating insult and the signaling pathways engaged in each model. Current evidence therefore supports IL-11 as a prominent but context-dependent amplifier of metabolic and alcohol-related liver injury, with the strongest human relevance currently provided by the concordant clinical and experimental findings in ALD.

### 4.3. From Impaired Regeneration to Maladaptive Repair

Effective hepatic repair requires timely attenuation of injury signals, re-entry of surviving hepatocytes into the cell cycle, and resolution of inflammatory and provisional ECM responses after tissue integrity is restored. When lost hepatocytes cannot be fully replaced, provisional ECM can temporarily stabilize injured regions and preserve tissue continuity. Under self-limited injury, this response subsides as the parenchyma is restored; when injury persists, however, the matrix may fail to resolve and gradually develop into a fibrotic scar. Repair then becomes maladaptive because structural stabilization is achieved without complete restoration of functional hepatocyte mass [4].

In hepatocytes, sustained IL-11 signaling maintains NOX4-dependent oxidative stress, ERK/JNK activation, mitochondrial dysfunction, and cell death while limiting the expression of PCNA, Cyclin D1, Ki67, and other regenerative markers [8,28,53,100]. The result is a tissue state in which continuing damage and incomplete proliferation coexist. By prolonging parenchymal loss while limiting its replacement, IL-11 may extend the requirement for scar-based repair and reduce the likelihood that the injury response will fully resolve.

The concept of IL-11 as a persistent pathological signal is further supported by aging models. Widjaja et al. showed that IL-11 progressively increases with age in the liver and other tissues, whereas deletion of Il11 or Il11ra1 and anti-IL-11 treatment initiated later in life ameliorate metabolic decline, frailty, and multiple age-associated phenotypes [102]. These findings suggest that IL-11 is not merely a transient stress mediator induced by severe exogenous injury; its progressive endogenous elevation may itself create a tissue environment that suppresses regeneration and sustains chronic stress.

IL-11 can therefore be viewed as a regulator of repair trajectory rather than simply as a pro-death or pro-proliferative cytokine. Its pathological significance lies in sustaining the mismatch between hepatocyte loss and replacement, thereby delaying the return to tissue homeostasis. Transient stress-associated IL-11 induction may accompany the early injury response, but when signaling persists, an initially adaptive matrix response may fail to resolve and progress toward fibrotic remodeling.

## 5. IL-11-Centered Cellular Crosstalk in the Transition to Liver Fibrosis

### 5.1. HSC-Autocrine IL-11 Signaling and Fibrogenic Activation

HSCs are the principal fibrogenic effector cells in the liver and the dominant source of collagen-producing myofibroblasts across different etiologies of chronic liver injury. In the healthy liver, quiescent HSCs store vitamin A and contribute to ECM homeostasis [103]. Following sustained parenchymal injury, they progressively become a major source of the hepatic myofibroblast pool and drive collagen deposition, tissue stiffening, and septal fibrosis [103,104]. The persistence or resolution of this activated phenotype therefore has a central influence on whether liver fibrosis progresses or regresses. Accordingly, identifying the signals that maintain HSC activation is essential for understanding the transition from transient repair to chronic fibrotic remodeling. IL-11 appears to function principally as one such reinforcing signal, sustaining the activated HSC phenotype after its initiation by parenchymal injury, inflammatory mediators, and altered tissue mechanics.

Studies in fibroblasts first identified IL-11 as a TGF-β1-induced autocrine mediator of myofibroblast activation [7,24]. Widjaja et al. subsequently demonstrated a comparable autocrine circuit in HSCs. TGF-β1 markedly induced IL11 transcription and IL-11 secretion in primary human HSCs and human precision-cut liver slices. HSCs expressed abundant IL-11Rα, and IL-11 stimulation activated ERK while increasing ACTA2 expression, collagen secretion, matrix invasion, and further IL-11 production. PDGF, CCL2, angiotensin II, and basic fibroblast growth factor also induced IL-11-dependent HSC activation, suggesting that IL-11 integrates several fibrogenic inputs into a shared autocrine response [25].

IL-11 additionally activates a gp130–SFK–YAP pathway that promotes HSC contraction, migration, mechanosensitive transcription, and collagen synthesis [57]. Its functional relevance is supported by complementary in vivo findings: species-matched rmIL-11 increased hepatic hydroxyproline and profibrotic gene expression, whereas Il11ra1 deletion and neutralizing antibodies reduced fibrosis in several dietary models of steatohepatitis [25]. HSC-directed IL-11 overexpression likewise aggravated CCl_4_-induced fibrosis, while IL-11 neutralization attenuated HSC activation and collagen accumulation [57].

IL-11 signaling also influences the persistence of HSC activation. Addition of IL-11- or IL-11Rα-neutralizing antibodies after stimulation with TGF-β1 or PDGF reduced ERK activity, ACTA2 expression, and collagen secretion despite continued exposure to the original stimulus [25]. In mice with established diet-induced fibrosis, IL-11 pathway blockade combined with withdrawal of the metabolic insult promoted matrix remodeling and fibrosis regression. These findings place HSC-autocrine IL-11 as a reinforcing and maintenance mechanism that stabilizes the fibrogenic phenotype while limiting its resolution.

### 5.2. IL-11-Centered Multicellular Crosstalk in Chronic Liver Disease

Parenchymal injury and fibrogenesis develop in parallel during chronic liver disease. In metabolic injury, lipid-loaded hepatocytes secrete IL-11, which acts locally to increase oxidative stress, mitochondrial dysfunction, and cell death, while also inducing ACTA2 and collagen expression in neighboring HSCs [28]. Hepatocyte-derived IL-11 therefore allows the severity of lipotoxic stress to influence the fibrogenic response before extensive tissue destruction has occurred. Similar communication may arise from other forms of injury. In *Schistosoma japonicum*- and CCl_4_-induced fibrosis, hepatocyte MCM7 promotes IL11 transcription through the SHCBP1–RACGAP1–STAT3 pathway, and IL-11 neutralization attenuates the associated HSC activation [56]. Thus, hepatocyte IL-11 production can be induced through distinct upstream mechanisms in different forms of chronic liver injury, transmitting parenchymal stress to the stromal compartment.

As HSC activation progresses, these cells become both a source and a target of IL-11. Their autocrine IL-11 activity maintains ERK- and SFK–YAP-dependent fibrogenic programs, while secreted IL-11 may extend the effect to surrounding hepatocytes. Conditioned medium from TGF-β1-activated LX-2 cells increased apoptosis and cleaved caspase-3 expression in LO-2 cells, whereas IL11 knockdown in LX-2 cells, IL-11 neutralization, or IL11RA knockdown in LO-2 cells reduced this response [48]. These findings suggest that activated HSCs can return an injurious signal to the parenchyma, although confirmation in primary cells and cell-specific in vivo models is still required. Continued hepatocyte injury would in turn maintain the release of IL-11 and other mediators capable of sustaining HSC activation.

The inflammatory compartment becomes increasingly involved as injury persists. IL-11 stimulates CCL2 production by HSCs, whereas IL-11Rα blockade reduces hepatic immune-cell accumulation, Ly6C-positive TGF-β-producing cells, and circulating TGF-β in experimental steatohepatitis [25]. HSC-derived IL-11 has also been shown to cooperate with IL-4 in promoting a profibrotic M2-like macrophage phenotype through PI3K–mTOR-dependent TGF-β synthesis and STAT3-associated chemokine production [57]. Macrophage depletion attenuated fibrosis induced by HSC-directed IL-11 overexpression, indicating that the resulting stromal response partly depended on macrophages. TGF-β and other mediators released in this setting can further stimulate HSC activation and IL-11 production, while HSC-derived chemokines sustain the recruitment and local activity of inflammatory cells.

The dominant cellular contribution is therefore likely to change over time. During early metabolic disease, IL-11 activity may be concentrated in stressed hepatocytes and primarily influence metabolism and survival. With progression to steatohepatitis, hepatocyte death, HSC activation, and macrophage recruitment become increasingly interdependent. Persistent HSC activation and matrix deposition subsequently stabilize the fibrotic microenvironment, allowing tissue remodeling to continue even as the relative contribution of the initiating insult changes. In advanced fibrosis and cirrhosis, these processes impair regeneration and alter vascular and immune homeostasis, providing a tissue context that can facilitate subsequent HCC development.

The strength of the available evidence nevertheless differs among these interactions. Hepatocyte-to-HSC communication and HSC-autocrine signaling are supported by primary-cell experiments together with genetic and antibody-based interventions in several mouse models [25,28]. Evidence for HSC-derived IL-11 acting on hepatocytes is based mainly on immortalized LX-2 and LO-2 cells [48], whereas its effects on macrophages have so far been examined in a limited number of fibrosis models [57]. Model dependence is also apparent: Il11ra1 deletion or antibody treatment reduced fibrosis in high-fat methionine- and choline-deficient (HFMCD) diet and Western diet–fructose models [25], but Il11ra1 deficiency had little effect on inflammation or collagen deposition in mice fed a choline-deficient, amino acid-defined high-fat diet [54]. IL-11 may therefore contribute substantially to multicellular amplification in selected settings without being equally important in every etiology or experimental model.

### 5.3. Extracellular Matrix Feedback and the Potential Involvement of Liver Sinusoidal Endothelial Cells (LSECs) and Cholangiocytes

ECM remodeling and altered tissue mechanics provide another layer of amplification within the IL-11-associated microenvironment. Progressive collagen deposition increases tissue stiffness and alters integrin, cytoskeletal, and YAP-dependent mechanotransduction in HSCs. Reanalysis of HSC transcriptomic data identified IL11 as one of the most strongly upregulated genes on stiff compared with soft substrates [25]. In parallel, IL-11 activates ERK- and gp130–SFK–YAP-dependent programs that promote HSC contraction, migration, and matrix production [25,57]. These observations suggest a feedback relationship in which matrix stiffening favors IL-11 expression and IL-11 further promotes matrix remodeling, although the requirement for IL-11 in stiffness-induced HSC activation has not yet been demonstrated directly in vivo.

Matrix remodeling also affects LSECs and cholangiocytes, whose communication with hepatic parenchymal, stromal, and immune compartments may influence local IL-11 production and responsiveness during vascular dysfunction and ductular reactions [105,106]. However, direct evidence remains limited. Endothelial cells outside the liver can express IL-11Rα and respond to IL-11 [107], but comparable functional evidence in LSECs is lacking. It is likewise unclear whether reactive cholangiocytes produce biologically relevant IL-11 or respond directly through IL-11Rα during cholestatic injury. Current evidence consequently defines the IL-11 crosstalk network most clearly among hepatocytes, HSCs, and macrophages, while LSECs and cholangiocytes represent biologically relevant but underexplored components. Cell-resolved expression studies, primary-cell experiments, spatial analyses, and cell-specific genetic interventions will be required to determine whether these cells participate directly in IL-11 signaling or are affected indirectly by remodeling of the inflammatory and fibrotic microenvironment.

## 6. IL-11-Centered Cellular Networks in HCC Progression and Tumor Microenvironment Remodeling

### 6.1. Malignant Phenotypes and Progression of HCC

HCC represents a major malignant outcome of chronic liver diseases arising from diverse etiologies. Although evidence remains insufficient to establish that IL-11 directly drives tumor initiation during the premalignant stage, an increasing number of clinical and experimental studies indicate that IL-11 contributes to the maintenance of proliferation, survival, migration, and invasion in established HCC and therefore serves as an important regulator of disease progression. Analyses of clinical specimens have shown that elevated IL-11 expression in HCC tissues is significantly associated with advanced tumor–node–metastasis (TNM) stage, shorter progression-free survival, and poorer overall survival following curative resection. IL-11 expression has also been identified as an independent predictor of adverse postoperative outcomes [62]. These findings establish the prognostic relevance of intratumoral IL-11 expression, although they do not determine whether its elevation is a cause or a consequence of aggressive tumor behavior.

Within HCC cells, IL-11 expression is controlled by multiple layers of post-transcriptional regulation and is linked to proliferative and prosurvival programs through the IL-11–IL-11Rα–STAT3 signaling axis. For example, miR-23b directly targets the 3′ untranslated region of IL-11, thereby suppressing the proliferation and clonogenic capacity of SMMC-7721 cells and inducing apoptosis. Restoration of IL-11 expression partially reverses these effects [60]. Similarly, the m^6^A-modified long noncoding RNA AC026356.1 interacts with the RNA-binding protein IGF2BP1 to enhance IL-11 mRNA stability, leading to increased IL-11 expression and secretion and consequently promoting HCC cell proliferation. Silencing IL-11 markedly attenuates this growth-promoting effect [59]. Direct manipulation of the IL-11 pathway has further confirmed its functional importance. In Hepa1-6 cells, silencing Il11ra significantly increases apoptosis and reduces the expression of the proliferation marker PCNA, whereas exogenous IL-11 promotes spheroid formation in three-dimensional cultures and enhances STAT3 phosphorylation [65]. Collectively, these findings support a tumor-cell-intrinsic role for the IL-11–IL-11Rα–STAT3 axis in sustaining HCC cell proliferation and survival.

In addition to regulating cell growth and survival, IL-11 signaling contributes to the migratory and invasive phenotypes of HCC cells. miR-124a-mediated downregulation of IL-11 not only inhibits HepG2 cell proliferation but also reduces cell migration and decreases the expression of the matrix metalloproteinases MMP2 and MMP9, suggesting that IL-11 may promote invasion by enhancing cell motility and facilitating extracellular matrix degradation [61]. Consistent with this observation, the AC026356.1–IGF2BP1 axis increases IL-11 expression and secretion by stabilizing IL-11 mRNA, thereby promoting HCC cell migration; this effect is markedly diminished following IL-11 silencing [59]. Moreover, the transmembrane trafficking protein TMED3 is upregulated in HCC tissues and portal vein tumor thrombi, and its high expression is associated with vascular invasion, metastatic propensity, and poor prognosis. TMED3 knockdown reduces IL-11 expression and STAT3 activation while suppressing HCC cell migration and invasion [63], further supporting the involvement of IL-11 signaling in HCC invasion and metastatic progression. Current mechanistic evidence therefore supports the contribution of IL-11 to established HCC, but it is mainly based on findings from individual cell lines and a limited range of experimental models. Confirmation using patient-derived systems and tumor-cell-specific perturbation in physiologically relevant HCC models remains limited.

### 6.2. Metastatic Colonization by Disseminated Tumor Cells

HCC metastasis is a multistep cascade involving local invasion, systemic dissemination, arrest at distant sites, metastatic colonization, and subsequent outgrowth [108]. The tumor-promoting effects of IL-11 extend to the later stages of this cascade, where it regulates the survival, adaptation, and expansion of disseminated tumor cells at secondary sites. The tumor-promoting role of IL-11 appears to be particularly relevant to the later stages of the metastatic cascade. Yuan et al. demonstrated that the TGF-β-induced long noncoding RNA lncRNA-ATB directly binds to and stabilizes IL-11 mRNA, thereby increasing autocrine IL-11 production and sustaining STAT3 activation in HCC cells. Depletion of IL-11 markedly impaired tumor cell colonization of the liver and lungs, while exerting little effect on epithelial–mesenchymal transition or local invasive capacity. These findings suggest that IL-11 does not primarily initiate early dissemination through EMT, but instead promotes the survival, adaptation, and clonal expansion of disseminated tumor cells after their arrival at secondary sites, thereby facilitating metastatic colonization and outgrowth [58]. Subsequent work on the AC026356.1–IGF2BP1 axis yielded consistent results. By stabilizing IL-11 mRNA, this regulatory pathway increases IL-11 secretion and promotes HCC cell proliferation, migration, and intrahepatic metastasis, whereas IL-11 silencing abolishes its prometastatic effects [59]. Together, these studies provide functional evidence that tumor-cell-derived IL-11 supports metastatic outgrowth, particularly after dissemination has occurred.

Clinical studies have likewise demonstrated that IL-11 expression in primary HCC is closely associated with the potential for distant metastasis, particularly bone metastasis. In an independent cohort of 350 patients undergoing curative hepatectomy, positive IL-11 expression in tumor tissues was significantly associated with advanced TNM stage and an increased risk of postoperative bone metastasis. After adjustment for tumor number, histological differentiation, vascular invasion, and other clinicopathological variables, IL-11 remained an independent risk factor for bone metastasis. Combined assessment of IL-11 and connective tissue growth factor (CTGF) further improved risk stratification for skeletal dissemination [49]. A subsequent study incorporated IL-11 together with vascular invasion, TNM stage, CXCR4, and CTGF into a clinicopathological prediction model and confirmed the value of intratumoral IL-11 for predicting postoperative bone metastasis in both training and validation cohorts [64]. Consistently, IL-11 is upregulated in bone-metastatic HCC tissues and highly metastatic HCC cell lines, accompanied by altered expression of CTGF, hypoxia-responsive molecules, and other metastasis-associated factors [50]. Together, these findings indicate that IL-11 not only supports the colonization of disseminated HCC cells in the liver and lungs but may also facilitate adaptation to specialized distant microenvironments, including bone, thereby promoting the establishment and sustained expansion of metastatic lesions.

### 6.3. Regeneration-Associated IL-11 Signaling and Post-Hepatectomy Recurrence

Curative hepatectomy remains a cornerstone of treatment for patients with resectable HCC, yet the high incidence of postoperative recurrence continues to limit long-term survival [109]. Partial hepatectomy initiates a tightly coordinated regenerative response involving inflammatory cytokines, growth factors, extracellular matrix remodeling, and activation of proliferative signaling pathways that restore liver mass and function [110]. Although essential for tissue repair, these regenerative signals may also provide a permissive environment for the survival and outgrowth of occult residual tumor cells [65,110]. In this context, IL-11 can be induced by oxidative stress and activate STAT3-dependent compensatory proliferation [45], providing a potential mechanistic link between the regenerative response of the remnant liver and postoperative HCC recurrence.

Clinical observations support this association. In a cohort of 290 patients with HCC who underwent curative hepatectomy, high intratumoral IL-11 expression was significantly associated with advanced TNM stage, shorter postoperative progression-free survival, and poorer overall survival, and was identified as an independent predictor of adverse outcomes [62]. Because IL-11 was measured in tumor tissue collected at resection, these findings establish a prognostic association but do not directly demonstrate that surgery-induced IL-11 contributes to recurrence. A more recent clinical study further reported that elevated IL-11 expression was associated with early postoperative recurrence and enhanced stromal remodeling in HCC [65], directly linking IL-11 upregulation to recurrence propensity, tumor stromal alterations, and unfavorable survival outcomes.

Mouse models of post-hepatectomy HCC recurrence have provided causal support for these clinical associations. Following liver resection, IL-11 and IL-11RA expression in the remnant liver increase in parallel with the regenerative response and are accompanied by activation of STAT3-mediated proliferative and prosurvival signaling. Genetic deletion of Il11ra significantly reduces postoperative tumor number and overall tumor burden, while decreasing the expression of PCNA, α-fetoprotein, and phosphorylated STAT3. Neutralization of IL-11 or pharmacological inhibition of STAT3 similarly suppresses postsurgical tumor outgrowth, providing complementary intervention-based evidence for this pathway [65]. Notably, Il11ra1 deletion reduced postoperative recurrence, whereas Il6 deletion did not significantly reduce postoperative tumor burden, suggesting that IL-11 has a relatively distinct role among IL-6 family cytokines in promoting HCC recurrence rather than reflecting a nonspecific shared family effect [66]. Overall, IL-11 appears to convert regenerative signals generated during liver repair into tumor-promoting signals that support the regrowth of residual HCC cells, making the IL-11 pathway a potential target for preventing postoperative recurrence. Direct causal evidence nevertheless remains concentrated in a single set of murine studies, and its relevance to postoperative recurrence in fibrotic or cirrhotic human livers requires further validation.

### 6.4. IL-11-Mediated Cellular Crosstalk in Tumor Microenvironment Remodeling

In addition to its direct effects on tumor cells, IL-11 participates in tumor microenvironment remodeling by regulating cellular crosstalk among malignant, stromal, and immune cells [10,111,112]. HCC typically develops in the context of chronic inflammation, hepatic fibrosis, or cirrhosis, in which activated HSCs and their myofibroblastic derivatives, excessive extracellular matrix deposition, myeloid-cell infiltration, and T-cell dysfunction collectively influence tumor growth, metastasis, and therapeutic response [113]. IL-11 can be produced by tumor cells, cancer-associated fibroblasts, myeloid cells, and other stromal populations. Through IL-11RA-gp130 signaling and downstream pathways, it may coordinate reciprocal interactions between tumor cells and their surrounding microenvironment, thereby establishing local conditions that favor tumor progression and immune evasion.

A stromal contribution is supported by myofibroblast-specific deletion of Msi2, which suppressed orthotopic HCC growth, intrahepatic and pulmonary metastasis, and prolonged survival in tumor-bearing mice. Msi2 deficiency inhibited ERK1/2 signaling in myofibroblasts and reduced IL-6 and IL-11 secretion, weakening their ability to promote HCC-cell proliferation, invasion, EMT, and cancer stem cell-like properties [51]. These findings implicate HSC-derived myofibroblasts in the paracrine support of HCC progression. However, because both cytokines were reduced and cytokine-specific rescue was not performed, the contribution attributable specifically to IL-11 remains incompletely resolved.

A more clearly defined multicellular interaction has been identified in the hepatic metastatic niche. In colorectal cancer liver metastasis, tumor-derived extracellular vesicles enriched in nidogen-1 activate HSCs and induce IL-11 secretion. The resulting IL-11 signaling promotes neutrophil recruitment and neutrophil extracellular trap formation, thereby creating a permissive hepatic niche for metastatic seeding and outgrowth. Treatment with an IL-11-neutralizing antibody reduces neutrophil infiltration, neutrophil extracellular trap formation, and liver metastatic burden [47]. The sequence from tumor-derived extracellular vesicles to HSC activation and subsequently to neutrophil recruitment provides direct evidence of communication across malignant, stromal, and immune cell populations, although its relevance to primary HCC remains to be established.

Evidence from HCC models further suggests that IL-11-dependent changes in the tumor microenvironment extend to adaptive immunity. The IL-11-neutralizing antibody 9MW3811 blocks formation of the IL-11–IL-11RA–gp130 signaling complex and suppresses the growth of Hepa1-6 syngeneic tumors. IL-11 blockade increases intratumoral infiltration and proliferation of CD8^+^ T cells, enhances cytotoxic effector programs, reduces features associated with T-cell exhaustion, and improves the therapeutic efficacy of anti-programmed cell death protein 1 (PD-1) treatment. Conversely, depletion of CD8^+^ T cells markedly attenuates the antitumor activity of IL-11 inhibition [67]. Single-cell analysis associated these effects with expansion of cytotoxic CD8^+^ T-cell populations and altered expression of XCL1 and CCL7. IL-11 blockade also increased PD-1 expression in an effector CD8^+^ T-cell subset, whereas adding 9MW3811 attenuated the exhaustion program observed after anti-PD-1 treatment alone. These complementary responses provide a mechanistic rationale for combining IL-11 inhibition with PD-1 blockade. Because 9MW3811 was administered systemically, however, these experiments do not identify the cellular source of the relevant IL-11 or the IL-11-responsive population responsible for the immune phenotype. The observed changes could reflect direct effects on immune cells, altered tumor-cell signaling, interruption of stromal support, or a combination of these mechanisms.

The hepatic disease background nevertheless complicates this interpretation. The 9MW3811 combination experiments were performed in subcutaneous transplant models without the chronic inflammation, fibrosis, vascular remodeling, and immune tolerance characteristic of most human HCCs. In mouse models of nonalcoholic steatohepatitis (NASH)-associated HCC, anti-PD-1 treatment increased resident-like CXCR6^+^CD8^+^PD-1^+^ T cells without inducing tumor regression and enhanced TNF-associated liver injury and hepatocarcinogenesis [114]. This setting separates the accumulation of PD-1-expressing T cells from productive antitumor immunity and indicates that the consequences of increasing CD8^+^ T-cell activity depend on the underlying liver disease. Combined IL-11 and PD-1 blockade should therefore be evaluated in orthotopic HCC models established in metabolic, viral, alcoholic, and fibrotic liver environments, with simultaneous assessment of tumor control, hepatic inflammation, fibrosis, and regenerative capacity.

Studies in other solid tumors suggest several cellular routes that could underlie the immune changes observed after IL-11 blockade in HCC. IL-11-STAT3 signaling can promote the generation of immunosuppressive myeloid cells, inhibit inflammatory cytokine production by CD4^+^ T cells, and reduce CXCL9 and major histocompatibility complex class I expression, thereby limiting CD8^+^ T-cell recruitment and tumor immune recognition [21,112,115]. These effects occur upstream of the PD-1 checkpoint and may limit the recruitment or antigen recognition of T cells that could subsequently be reinvigorated by checkpoint blockade. In brain metastases from EGFR-mutant non-small-cell lung cancer, reactive astrocyte-derived IL-11 was reported to increase tumor-cell programmed death-ligand 1 (PD-L1) expression through IL-11Rα/gp130- and EGFR–AKT-dependent signaling and to promote T-cell apoptosis [116]. In lung adenocarcinoma, IGF2BP1-dependent stabilization of m^6^A-modified IL11 mRNA increased IL-11 production and impaired CD8^+^ T-cell proliferation, cytotoxicity, and secretion of TNF-α, IFN-γ, and IL-2 [80]. These studies suggest that IL-11 may influence checkpoint responses through both tumor-cell PD-L1 regulation and suppression of effector T-cell function, although neither directly evaluated IL-11 blockade together with PD-1 inhibition.

Preliminary findings presented at the 2026 American Association for Cancer Research (AACR) Annual Meeting extend this association to melanoma. Age-related increases in IL-11 were reported to promote accumulation of polymorphonuclear myeloid-derived suppressor cells (PMN-MDSCs), reduce CD8^+^ T-cell infiltration, and impair responses to anti-PD-1 therapy. IL-11 blockade improved anti-PD-1 efficacy and produced complete tumor responses in aged melanoma-bearing mice [117]. These findings introduce age-dependent myeloid polarization as another potential mechanism connecting IL-11 to checkpoint resistance. However, the evidence is currently available only as a conference abstract and awaits full methodological reporting and independent validation. Although these cross-cancer findings provide mechanistic support for the potential immunosuppressive role of IL-11 in HCC, direct validation in orthotopic HCC models arising in fibrotic livers and in clinical HCC specimens remains necessary.

Collectively, current evidence supports several IL-11-dependent interactions within the hepatic tumor microenvironment rather than a single uniformly defined pathway. The principal unresolved issue is the spatial and cellular context in which IL-11 signaling becomes functionally important. Cell-type-specific perturbation and spatial profiling in orthotopic HCC arising in fibrotic or cirrhotic livers will be required to distinguish direct responses to IL-11 from secondary consequences of altered tumor or stromal activity. Such studies should also identify the patients most likely to benefit and determine whether IL-11 blockade can enhance immune checkpoint inhibition without compromising hepatic repair or aggravating the underlying liver disease. Until these questions are resolved, this combination should be regarded as a promising but unvalidated preclinical strategy.

### 6.5. From Liver Injury and Fibrosis to HCC: Integration of IL-11-Centered Cellular Crosstalk

Taken together, current experimental evidence supports an integrated model in which IL-11-centered cellular crosstalk amplifies hepatocyte injury, sustains fibrotic remodeling, and promotes HCC progression. During acute toxic injury, stressed hepatocytes induce an autocrine IL-11 circuit that amplifies mitochondrial dysfunction and cell death while restricting regenerative cell-cycle re-entry. If the insult resolves, this response may subside; if injury is severe, repeated, or persistent, continued hepatocyte loss and incomplete replacement prolong provisional matrix deposition and favor maladaptive repair [8,53]. Comparable responses in metabolic and alcohol-related liver diseases further position hepatocyte-autocrine IL-11 signaling as a shared amplifier of liver injury across distinct etiologies [25,28,55].

Persistent or recurrent liver injury prolongs the repair response and increases communication among hepatocytes, inflammatory cells, and HSCs. Across different etiologies, IL-11 can help direct this response toward chronic remodeling: hepatocyte-derived IL-11 transmits parenchymal stress to HSCs, whereas HSC-autocrine IL-11 maintains their contractile and matrix-producing phenotype. HSC-derived chemokines recruit macrophages, whose profibrotic mediators further reinforce stromal activation [25,57]. Continued ECM deposition and matrix stiffening can then sustain HSC activation, transforming temporary tissue stabilization into persistent fibrosis.

In advanced fibrosis and cirrhosis, activated HSCs, rigid ECM, myeloid-cell accumulation, and impaired T-cell function create a tissue environment permissive for HCC. IL-11 has not been established as a direct initiator of malignant transformation, but established HCC can exploit this pre-existing network. Tumor-cell-autocrine IL-11–STAT3 signaling supports survival and expansion, while stromal and immune interactions further promote tumor progression [58,59,65]. IL-11 also appears particularly relevant after dissemination: it promotes the survival and outgrowth of tumor cells at secondary sites and is clinically associated with postoperative bone metastasis, supporting a role in metastatic colonization rather than exclusively in local invasion [49,50,64]. IL-11 blockade can additionally increase cytotoxic CD8^+^ T-cell activity and enhance PD-1 inhibition in preclinical HCC models [67].

**Figure 4:**
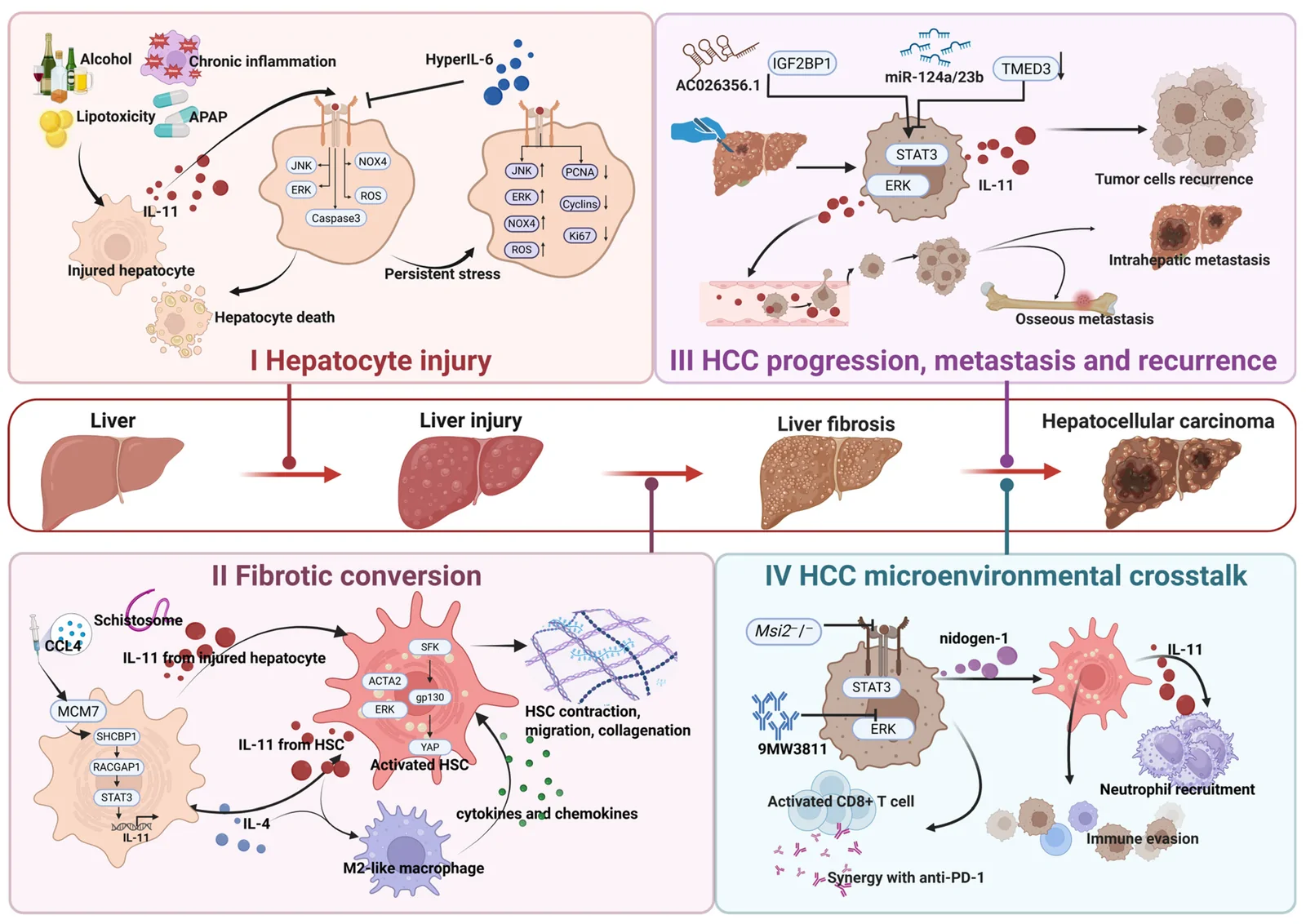
IL-11: a critical pathogenic nexus in liver disease.

Hepatectomy superimposes acute tissue injury and regenerative demand on a liver that often already contains fibrosis or cirrhosis. The resulting increase in IL-11–IL-11Rα–STAT3 activity in the remnant liver supports the expansion of occult residual tumor cells, linking postoperative repair to recurrent tumor growth. Genetic or pharmacological inhibition of this pathway reduces postoperative tumor burden and recurrence in mouse models [65]. Overall, interconnected IL-11-dependent autocrine and paracrine circuits amplify hepatocyte injury, maintain stromal and inflammatory activation, and support tumor growth, metastatic colonization, immune escape, and postoperative recurrence. These interconnected cellular circuits and their evolution across liver disease stages are summarized in Figure 4. The principal IL-11-associated signaling pathways and their functional consequences in liver disease are summarized in Table 2.

## 7. Translational Potential of IL-11-Targeted Therapy

### 7.1. Therapeutic Strategies Targeting the IL-11/IL-11RA Axis

As the pathogenic roles of IL-11 in chronic liver injury, fibrosis progression, and remodeling of the HCC immune microenvironment have become increasingly evident, therapeutic development targeting IL-11 signaling has shifted from the early paradigm of exogenous IL-11 supplementation toward inhibition of pathological IL-11 signaling. Current strategies for targeting the IL-11 pathway can be broadly divided into four categories: direct neutralization of IL-11 or blockade of IL-11RA, nucleic acid-based suppression of IL-11 signaling, engineered IL-11 antagonists and peptide-based inhibitors, and IL-11RA-directed targeting and payload delivery.

#### 7.1.1. Neutralization of IL-11 or Blockade of IL-11RA

Direct neutralization of IL-11 or blockade of IL-11RA currently represents the best-characterized therapeutic approach. Representative preclinical reagents include the IL-11-neutralizing antibodies X203 and MAB218 and the IL-11RA-blocking antibody X209, whereas 9MW3811, BI 765423, and LASN01 have entered clinical development. In acetaminophen-induced liver injury, X209 reduced hepatocyte death and suppressed NOX4, ERK, and JNK activation, while restoring PCNA, cyclins, and retinoblastoma protein phosphorylation. Importantly, delayed treatment remained effective, improved survival after lethal acetaminophen exposure, and enhanced the recovery of selected regenerative markers when combined with N-acetylcysteine [8]. These findings suggest that IL-11RA blockade may both limit ongoing hepatocellular injury and facilitate regenerative recovery. In experimental steatohepatitis, X203 and X209 reduced hepatic fibrosis, steatosis, inflammation, serum ALT levels, and ERK activation across several dietary models [25]. X209 additionally improved glucose and lipid abnormalities in the Western diet with liquid fructose model. In mice with established fibrosis, combining metabolic stimulus withdrawal with IL-11 pathway blockade accelerated collagen regression, with a numerically greater effect observed for X209. Sustained antibody administration did not cause detectable abnormalities in platelet counts or the principal serum biochemical indices examined, providing preliminary evidence of tolerability in mice [25]. In alcohol-related liver disease, IL-11RA blockade reduced ethanol-induced pathological signaling, hepatocyte injury, and hepatic inflammation [55]. The available human evidence is translational rather than therapeutic: X209 suppressed injury-associated signaling in primary human hepatocytes, IL-11-dependent fibrogenic responses were demonstrated in primary human HSCs and human precision-cut liver slices, and circulating and hepatic IL-11 levels were associated with disease severity and transplant-free survival in patients with alcohol-related liver disease [8,25,55]. These findings support the relevance of the target to human liver disease but do not establish clinical efficacy.

In the context of cancer therapy, the distinguishing feature of 9MW3811 lies in its combination with immunotherapy. In the Hepa1-6 hepatoma model, 9MW3811 inhibits tumor growth, and combination with anti-PD-1 further enhances antitumor efficacy, accompanied by increased CD8^+^ T cell infiltration and proliferation, attenuated T cell exhaustion, upregulation of XCL1 and effector molecules, and reduced CCL7 [67]. These findings suggest that IL-11 neutralization may improve antitumor immunity and increase responsiveness to immune checkpoint inhibition. The therapeutic potential of IL-11 neutralization has also been demonstrated in colorectal cancer liver metastasis. NID1-enriched tumor-derived extracellular vesicles activated hepatic stellate cells and induced IL-11 secretion, leading to neutrophil recruitment and neutrophil extracellular trap formation. Administration of MAB218 reduced neutrophil extracellular trap (NET) formation and metastatic burden, indicating that IL-11 blockade may disrupt stromal–myeloid interactions required for the establishment of a permissive hepatic metastatic niche [47]. These studies identify immunotherapy sensitization and metastatic-niche disruption as two potential oncological applications of IL-11 blockade, although neither has been evaluated therapeutically in patients with HCC or liver metastases.

Beyond the preclinical evidence discussed above, several IL-11- or IL-11RA-blocking antibodies have advanced into clinical trials, where they are primarily being evaluated for safety and preliminary efficacy in non-hepatic indications. The progress and translational hurdles for applying these agents to liver disease will be further addressed in Section 7.2.1.

#### 7.1.2. Nucleic Acid-Based Suppression of IL-11 Signaling

Nucleic acid-based approaches provide an alternative strategy for suppressing IL-11 signaling by either reducing the expression of IL11 or IL11RA or enabling local production of IL-11-neutralizing biologics. In this context, nanoparticle and lipid-based systems function primarily as delivery platforms that improve tissue exposure, cellular selectivity, and duration of target engagement rather than as the therapeutic mechanism itself.

Direct gene silencing has been evaluated using activated hepatic stellate cell-targeted nanoparticles. Zhang et al. developed NP-AEAA to deliver small interfering RNAs (siRNAs) against either Il11 or Il11ra1 to activated HSCs. In two mouse models of MASH, both formulations preferentially accumulated in activated HSCs, reduced IL-11/ERK signaling and HSC activation, and attenuated steatosis, inflammation, and fibrosis [118]. Notably, siIl11ra1@NP-AEAA produced greater improvements in several hepatic endpoints than siIl11@NP-AEAA, suggesting that receptor-level silencing may provide more complete pathway suppression by preventing responses to IL-11 regardless of its cellular source. These findings establish cell-selective RNA interference as a feasible means of disrupting pathological IL-11 signaling within the fibrotic liver.

A complementary approach uses mRNA delivery to convert the liver into a local source of an IL-11-neutralizing biologic. Using an AA3G-based lipid nanoparticle, the same group delivered mRNA encoding an IL-11-neutralizing single-chain variable fragment, resulting in sustained intrahepatic production of the neutralizing antibody fragment and inhibition of IL-11 signaling in hepatocytes and HSCs [119]. This strategy prevented or reversed pathological features in both early-stage and fibrosis-associated MASH models and showed greater efficacy than systemic administration of the corresponding recombinant single-chain variable fragment (scFv) protein, consistent with prolonged local antibody production and enhanced hepatic exposure. Rather than permanently altering IL11 or IL11RA expression, this approach provides transient and potentially titratable local neutralization through repeated mRNA administration.

Together, these studies demonstrate two mechanistically distinct nucleic acid strategies for IL-11 inhibition: direct silencing of the ligand or receptor and local expression of an IL-11-neutralizing biologic. Both approaches have so far been evaluated only in preclinical liver disease models. Their clinical translation will require confirmation that the cellular selectivity and hepatic distribution observed in mice are retained in the heterogeneous and structurally distorted livers of patients with advanced MASH or cirrhosis. Additional challenges include endosomal escape, durability of target suppression, off-target gene silencing, innate immune activation, repeated-dose tolerability, and scalable manufacturing. For mRNA-mediated antibody expression, the magnitude and duration of intrahepatic protein production and the immunogenicity of both the delivery vehicle and encoded product will also require careful evaluation. Nevertheless, nucleic acid-based approaches may offer an important advantage over systemic blockade by enabling spatially restricted and potentially tunable suppression of IL-11 signaling within the diseased liver.

#### 7.1.3. Engineered IL-11 Antagonists and Peptide-Based Inhibitors

Engineered IL-11 variants can inhibit signaling by retaining IL-11RA binding while disrupting formation of a signaling-competent receptor complex. Early studies identified W147A as an IL-11 antagonist, but subsequent structural analyses showed that W147A alone is insufficient for robust inhibition. In IL-11Δ10/Mutein, W147A combined with AB-loop substitutions permits initial IL-11RA/gp130 engagement but prevents assembly of the active hexamer, thereby competitively inhibiting IL-11 signaling [17]. These findings highlight the need to exclude residual agonistic activity during the development of engineered cytokine antagonists.

Subsequent studies have extended engineered IL-11 antagonism from structural and cellular characterization to in vivo disease models. An engineered high-affinity IL-11 decoy cytokine inhibited receptor signaling and proliferation and delayed tumor progression in a mouse model of lung adenocarcinoma [120]. IL-11 Mutein has also been used therapeutically in colorectal cancer models, where it increased CXCL9 and MHC-I expression, enhanced CD8^+^ T-cell infiltration, and suppressed tumor growth [112]. More recently, site-specific dimerization of IL-11 W147C generated a receptor-biased antagonist that largely preserved IL-11RA binding while markedly reducing gp130 engagement. The resulting dimer inhibited fibroblast activation, prolonged systemic exposure, and attenuated bleomycin-induced pulmonary fibrosis in mice [121]. Pang et al. further developed the recombinant antagonist IL-11-6M and a site-specifically PEGylated long-acting derivative, analogue 13, which retained high-affinity IL-11RA binding and antagonistic activity and showed therapeutic efficacy in unilateral ureteral obstruction-induced renal fibrosis [122]. These studies establish that engineered IL-11 ligands can be converted into pharmacologically active antagonists with efficacy across cancer and fibrotic disease models.

Peptide-based inhibitors provide a complementary strategy for directly disrupting IL-11–receptor interactions using smaller molecular scaffolds. Lear et al. identified cyclic peptides against IL-11 and IL-11RA by phage-display screening. Peptide 4 inhibited IL-11–IL-11RA association, and subsequent optimization generated peptide 15 with improved biochemical inhibitory activity, although these compounds were evaluated primarily in binding assays [123]. More recent studies have advanced this approach into cellular and in vivo validation. The macrocyclic peptide 4L2, identified through the random nonstandard peptide integrated discovery platform, bound IL-11 with nanomolar affinity, and its optimized derivative 4L2-P13D showed enhanced antagonistic activity and significant antifibrotic effects in experimental renal fibrosis [124]. In 2026, an alternative design strategy generated the IL-11-derived cyclic peptide P19 from the receptor-interacting C-terminal region of IL-11. P19 inhibited IL-11 signaling, reduced ERK1/2 phosphorylation, and attenuated renal fibrosis in vivo without obvious tissue toxicity in mice [125].

Collectively, engineered cytokine antagonists and peptide-based inhibitors have progressed from receptor-structure and binding studies to therapeutic proof of concept in cancer, pulmonary fibrosis, and renal fibrosis. However, these modalities remain at the preclinical stage, and none has yet been directly validated as a therapeutic intervention in experimental liver disease or evaluated clinically. Further development will require optimization of pharmacokinetics, tissue exposure, proteolytic stability, immunogenicity, and dosing durability, together with rigorous exclusion of residual agonistic activity. These approaches therefore broaden the range of direct IL-11 inhibitors beyond monoclonal antibodies but remain substantially less mature than antibody-based blockade.

#### 7.1.4. IL-11RA-Directed Targeting and Payload Delivery

IL-11RA can also be exploited as a cell-surface molecular address for selective therapeutic delivery rather than as a target for direct inhibition of IL-11 signaling. One of the earliest examples is BMTP-11, an IL-11RA-binding cytotoxic peptidomimetic that induced apoptosis and suppressed tumor growth in IL-11RA-positive non-small-cell lung cancer models [126]. This strategy subsequently entered a first-in-human exploratory study in six patients with metastatic castration-resistant prostate cancer, in which BMTP-11 localized to IL-11RA-positive bone metastases and triggered apoptotic tumor-cell death, but the trial was limited by dose-limiting nephrotoxicity and did not demonstrate clinical efficacy [127]. IL-11RA-directed chimeric antigen receptor T (CAR-T) cells selectively killed IL-11RA-positive osteosarcoma cells and induced regression of established pulmonary metastases in mice, although this approach remains at the preclinical stage [128].

The same receptor-targeting principle has been extended to radionuclide and nanoscale drug delivery. The radiolabeled cyclic peptide ^153^Sm-DTPA-c(CGRRAGGSC) selectively bound IL-11R-expressing MHCC97-H human liver cancer cells, accumulated in xenografted tumors, and inhibited tumor growth in vivo [129], providing direct proof of concept for IL-11RA-guided therapeutic delivery in liver cancer. IL-11RA-binding motifs have also been incorporated into nanocarriers. For example, IL11-PDox, an IL-11RA-targeted redox-responsive polymersome carrying doxorubicin, enhanced drug delivery to IL-11RA-high osteosarcoma cells and suppressed primary tumor growth, recurrence, and pulmonary metastasis, with additional activity demonstrated in patient-derived tumor models [130]. Subsequent studies have further incorporated IL-11/IL-11RA recognition into cell-membrane-coated nanoparticles and multifunctional liposomal systems [131,132], indicating that this receptor-targeting principle is adaptable to different therapeutic payloads and delivery platforms.

These approaches are mechanistically distinct from IL-11 or IL-11RA neutralization because their therapeutic effects depend primarily on receptor-guided payload delivery. Translation to liver disease will require careful assessment of IL-11RA expression heterogeneity and potential on-target, off-tumor toxicity, particularly because IL-11RA is also expressed by nonmalignant hepatic cells. Although IL-11RA-directed targeting has progressed from preclinical models to limited human target-engagement studies, its therapeutic utility in disease-relevant HCC or chronic liver disease remains to be established.

In addition, small molecules and natural compounds may complement these IL-11-specific approaches by modulating downstream or cooperating pathways, including STAT3, ERK/JNK, NOX4–ROS, and mTOR-associated signaling [7]. Because these signaling nodes are shared by multiple cytokines and cellular stress responses, their inhibition is generally less selective than ligand neutralization, receptor blockade, or RNA-based silencing and may therefore be better suited to combination therapy. In fibrotic liver disease, where IL-11 signaling interacts with TGF-β activity, oxidative stress, lipotoxicity, and inflammatory circuits, combining IL-11 blockade with metabolic, antioxidant, antifibrotic, or immunomodulatory interventions may more effectively suppress hepatocyte injury, HSC activation, and inflammatory-cell recruitment, thereby improving control of chronic liver injury and potentially reducing the risk of hepatocarcinogenesis.

### 7.2. Clinical Translation of IL-11/IL-11RA-Targeted Therapy in Liver Disease

#### 7.2.1. Clinical Development and the Gap to Liver Indications

Clinical exposure to pharmacological IL-11 in liver disease predates current IL-11-blocking strategies. A small open-label study in chronic HCV reported reduced hepatic inflammatory activity and serum ALT after rhIL-11 treatment, although fibrosis did not significantly improve and HCV RNA increased [46]. Clinical development of IL-11 pathway-blocking agents has so far proceeded primarily through systemic antibodies in non-hepatic indications. As of August 2026, the IL-11-neutralizing antibody BI 765423 had completed phase I evaluation in healthy participants and entered a recruiting phase IIa trial in idiopathic pulmonary fibrosis (IPF) [133,134]. The IL-11RA-blocking antibody LASN01 has provided the most detailed published human data. Phase I studies in healthy participants and small pulmonary fibrosis and thyroid eye disease (TED) cohorts showed sustained systemic exposure, inhibition of ex vivo IL-11-induced STAT3 phosphorylation, and no treatment-related serious or severe adverse events. In a small placebo-controlled phase II trial in thyroid eye disease, 88% of LASN01-treated participants achieved a clinical activity score of 0 or 1 compared with 44% receiving placebo, whereas improvement in proptosis was limited [135]. The IL-11-neutralizing antibody 9MW3811 has completed phase I evaluation in healthy participants and entered a recruiting phase II trial for pathological scars, although peer-reviewed clinical results have not been reported [136,137]. Outside pathway blockade, the phase 0 study of BMTP-11 provides limited first-in-human evidence that IL-11RA can function as a therapeutic target [127], although the study established target localization and biological activity rather than clinical antitumor efficacy. These programs demonstrate that systemic IL-11 or IL-11RA blockade can be advanced into human testing.

However, this experience cannot yet be regarded as clinical validation of IL-11 blockade in liver disease. The populations studied have consisted of healthy participants or patients with non-hepatic fibro-inflammatory disorders, and no clinical trial has yet evaluated therapeutic IL-11 or IL-11RA blockade in patients with liver disease. Differences in disease etiology, stage, hepatic functional reserve, and treatment setting may substantially influence the required dose, treatment duration, therapeutic window, and acceptable risk. Current clinical experience therefore reduces uncertainty surrounding the therapeutic modality but does not establish a development framework for hepatic indications. The major IL-11/IL-11RA-targeted therapeutic strategies discussed above, together with their mechanisms, current stages of development, available evidence, and principal translational limitations, are summarized in Table 3.

#### 7.2.2. Safety Considerations and Therapeutic Window

Safety is a prerequisite question that must be addressed before IL-11-targeted therapies can advance into clinical application for liver diseases. The available evidence is reassuring but remains limited. Sustained IL-11 or IL-11RA blockade in mouse models did not produce detectable abnormalities in platelet counts or the principal serum biochemical indices examined [25]. In aged mice, 25 weeks of anti-IL-11 treatment improved metabolic and functional measures, while treatment continued until death was associated with extended lifespan without an overt toxicity signal [102]. Early clinical experience with LASN01 likewise showed no treatment-related serious or severe adverse events during exposure extending to approximately 12 months [135]. However, the small study populations, limited number of treated participants, and predominance of non-hepatic indications preclude assessment of uncommon, delayed, or liver-specific adverse effects.

Potential risks should be considered in relation to the broader biology of IL-11. IL-11 signaling has been implicated in hematopoietic regulation, skeletal and reproductive biology, and context-dependent tissue remodeling [7]. Nevertheless, developmental phenotypes associated with lifelong pathway deficiency and the thrombopoietic effects of pharmacological recombinant IL-11 should not be assumed to predict the consequences of IL-11 blockade initiated in adults. Current evidence does not establish thrombocytopenia or impaired tissue repair as class-specific toxicities of IL-11-targeted agents. Long-term studies should nonetheless monitor blood-cell and platelet counts, coagulation, infection, bone and reproductive health, wound healing, infusion reactions, immunogenicity, and the durability of pathway suppression.

The therapeutic window may be narrower in patients with impaired hepatic reserve, who are more susceptible to infection, bleeding, renal dysfunction, malnutrition, and clinical decompensation [138]. Safety is therefore likely to depend on disease stage, treatment duration, concomitant therapy, and the timing of pathway inhibition relative to acute deterioration or surgery. Clinical studies should incorporate predefined criteria for dose modification or treatment interruption and monitor hepatic function, portal-hypertension-related complications, renal function, infection, and recovery after tissue injury. Nucleic acid-based liver-directed approaches may reduce systemic exposure but introduce additional risks related to biodistribution, hepatic accumulation, carrier toxicity, innate immune activation, off-target silencing, and the duration of transgene expression or target suppression. IL-11RA-directed payload-delivery strategies raise a different set of safety concerns, particularly receptor-dependent on-target, off-tumor toxicity in normal IL-11RA-expressing tissues, together with payload-specific toxicities associated with cytotoxic drugs, engineered immune cells, or radionuclides. Thus, the safety of each IL-11-targeted modality must be established separately rather than inferred solely from target specificity or early experience with systemic antibodies.

#### 7.2.3. Biomarker-Guided Patient Selection and Pharmacodynamic Assessment

Biomarker requirements are likely to differ according to therapeutic mechanism. For IL-11 pathway-blocking approaches, clinical translation will require identifying patients with biologically active and therapeutically relevant IL-11 signaling rather than assuming uniform pathway dependence within a diagnostic category. Circulating IL-11 alone is unlikely to provide an adequate selection biomarker because IL-11 acts predominantly within local tissue microenvironments and may be present at low or variable concentrations in peripheral blood [7]. Hepatic IL11 or IL11RA expression indicates pathway availability but does not by itself establish functional signaling activity or therapeutic dependence. Baseline enrichment may therefore require integration of ligand and receptor expression with the abundance and spatial distribution of IL-11-responsive cell populations and downstream signaling features such as ERK/JNK–NOX4 activation.

By contrast, IL-11RA-directed payload-delivery strategies depend primarily on receptor accessibility rather than on active IL-11 signaling. For these approaches, quantitative cell-surface IL-11RA expression, receptor internalization, spatial heterogeneity, and the expression differential between diseased and normal tissues may be more relevant than circulating IL-11 concentrations or downstream pathway activation. This distinction is particularly important in HCC, where therapeutic selectivity will depend not only on IL-11RA abundance within malignant tissue but also on receptor expression in surrounding nonmalignant hepatic cells.

Pharmacodynamic assessment should likewise be adapted to therapeutic modality. For pathway-blocking agents, measurements should include systemic exposure, anti-drug antibodies where applicable, and direct or indirect evidence of pathway inhibition. The ex vivo IL-11-induced STAT3 phosphorylation assay used in the LASN01 clinical program provides one example of peripheral target-engagement assessment [135], although it does not demonstrate inhibition of intrahepatic signaling. When tissue sampling is feasible, paired evaluation of IL-11-responsive signaling, cellular activation, and fibrosis-related changes before and during treatment would provide stronger evidence of hepatic target engagement. For IL-11RA-directed delivery platforms, pharmacodynamic assessment should instead establish receptor-dependent localization, internalization, payload delivery, and tumor-to-normal tissue distribution.

No individual biomarker currently has sufficient specificity or clinical validation to serve as a companion diagnostic for IL-11-targeted therapy. Liver biopsy provides the most direct molecular information but is limited by invasiveness, sampling variability, and spatial heterogeneity. Early clinical studies should therefore combine blood-based, tissue-based, and imaging measurements and distinguish biomarkers used for patient selection, target engagement, and treatment response. Candidate biomarkers should initially support prespecified stratification and exploratory response analyses rather than mandatory enrollment thresholds. Subsequent studies must determine whether a given marker predicts benefit from IL-11 pathway inhibition, identifies suitability for IL-11RA-directed delivery, merely reflects disease severity, or functions only as a pharmacodynamic indicator.

#### 7.2.4. Clinical Trial Design and Remaining Translational Challenges

Clinical development should account for differences in disease etiology, tempo, reversibility, hepatic functional reserve, and therapeutic intent rather than applying a uniform trial design across liver diseases. Early-phase studies should define biologically active and pharmacologically relevant doses using pharmacokinetic and target-engagement measurements rather than relying exclusively on the maximum tolerated dose. Treatment duration and assessment schedules should reflect the expected kinetics of the disease process and the proposed mechanism of benefit. Because hepatic dysfunction may alter drug exposure, physiological tolerance, and susceptibility to adverse events, dose escalation should be stratified according to hepatic reserve and accompanied by predefined interruption and stopping criteria.

Clinical endpoints should distinguish pathway engagement, modification of disease activity or tissue remodeling, and patient-level clinical benefit. For pathway-blocking therapies, systemic exposure alone would be insufficient without evidence that IL-11 signaling is effectively suppressed, particularly within the liver. Conversely, changes in nonspecific markers of injury or fibrosis would not by themselves establish that a therapeutic effect was mediated through IL-11 inhibition. Early studies should therefore combine pharmacodynamic measurements with standardized biochemical, imaging, histological, functional, and clinical outcomes appropriate to the intended indication. For IL-11RA-directed payload-delivery strategies, proof of selective receptor-mediated localization and adequate tumor-to-normal tissue discrimination should precede interpretation of antitumor efficacy.

Translation from preclinical models to human liver disease remains a major challenge. The species-dependent pharmacology observed with recombinant IL-11 illustrates how receptor binding does not necessarily predict equivalent downstream signaling or biological activity across species [8,24]. Candidate pathway-blocking agents should therefore be evaluated in species-matched systems and complemented by primary human hepatocytes and HSCs, multicellular co-cultures, liver organoids, precision-cut liver slices, and patient-derived tumor models where appropriate. These platforms may help determine which cell populations respond directly to IL-11 inhibition and whether therapeutic effects are retained within the inflammatory, metabolic, fibrotic, or malignant microenvironment relevant to the intended indication.

Development requirements will also differ substantially among therapeutic modalities. Systemic antibodies require characterization of receptor occupancy, tissue penetration, immunogenicity, and the consequences of prolonged target suppression. Nucleic acid-based approaches require detailed assessment of biodistribution, endosomal escape, carrier-related toxicity, innate immune activation, expression or silencing duration, manufacturing consistency, and reversibility [118,119]. Engineered IL-11 antagonists and peptide-based inhibitors introduce additional requirements related to residual agonism, proteolytic stability, pharmacokinetics, tissue exposure, and immunogenicity. IL-11RA-directed payload-delivery platforms instead require quantitative assessment of receptor density and internalization, tumor-to-normal tissue distribution, and payload-specific toxicity; radionuclide-based approaches additionally require appropriate dosimetry.

Clinical translation should therefore proceed through modality-specific development programs rather than assuming that safety or efficacy can be transferred across different approaches to the IL-11/IL-11RA axis. Combination regimens should generally be pursued only after adequate exposure, target engagement, and safety have been established for the individual IL-11-targeted modality. The first liver-directed trials should establish a clear, modality-appropriate sequence of evidence: adequate exposure and target engagement, demonstrable pathway inhibition for signaling-blocking approaches or selective payload delivery for IL-11RA-directed platforms, a measurable biological response, and preservation of hepatic function. Larger efficacy studies and more complex combinations should follow only after these requirements have been met.

## 8. Conclusions

The interpretation of IL-11 in liver disease has changed substantially. Early pharmacological studies using rhIL-11 in rodents suggested anti-inflammatory and hepatoprotective effects, and limited clinical evidence in chronic HCV also suggested a short-term anti-inflammatory effect of rhIL-11 in humans. Subsequent recognition of species-dependent ligand activity showed that the rodent findings could not be assumed to represent endogenous IL-11 biology. Studies using species-matched ligands, genetic perturbation, and neutralizing antibodies have since predominantly associated endogenous IL-11 with hepatocyte injury, impaired regeneration, and fibrotic remodeling [8,24,25]. Consequently, the field has shifted from examining the effects of exogenous rhIL-11 toward defining the cellular sources, signaling mechanisms, and pathological functions of endogenous IL-11 within the liver.

At the tissue level, IL-11 operates through interconnected parenchymal, stromal, immune, and tumor-cell circuits. In hepatocytes, stress-induced IL-11 reinforces oxidative and mitochondrial dysfunction, metabolic disturbance, and cell death while limiting effective regeneration. This parenchymal response is transmitted to HSCs through IL-11 and other injury-associated signals. Once activated, HSCs maintain IL-11-dependent autocrine signaling, produce ECM, and secrete chemokines that recruit macrophages; macrophage-derived mediators and increasing matrix stiffness then further reinforce stromal activation [25,57]. IL-11 therefore not only acts within individual cell populations but also coordinates reciprocal interactions that allow hepatocyte injury and incomplete repair to sustain inflammation and fibrosis. The resulting fibrotic and immune microenvironment can subsequently support HCC progression. Tumor-cell-autocrine IL-11–STAT3 signaling promotes survival and expansion, whereas communication with stromal and immune cells facilitates immune escape and tumor outgrowth [58,59,67]. IL-11 also supports the colonization of disseminated tumor cells and may enable residual tumor cells to exploit regenerative signals after hepatectomy, thereby contributing to metastasis and postoperative recurrence [49,50,64,65]. These observations place IL-11 at the intersection of hepatocyte injury, maladaptive repair, fibrotic remodeling, and the progression of established HCC, without implying that it initiates the underlying liver disease or malignant transformation.

Important uncertainties remain. Species mismatch explains much of the disagreement with early pharmacological studies but does not fully resolve reports suggesting context-dependent beneficial effects of IL-11 or limited consequences of pathway disruption in some experimental settings [45,52,54]. The influence of injury context, sex, treatment timing, and the balance between STAT3 and ERK/JNK signaling requires further clarification. The cellular basis of IL-11-mediated immune remodeling and the efficacy and safety of combining IL-11 inhibition with immunotherapy in chronically injured livers also remain uncertain [67,114]. A substantial proportion of the mechanistic and interventional evidence has emerged from a relatively limited number of research groups, while human evidence in liver disease remains mainly associative or translational, and clinical experience with IL-11 pathway blockade is currently confined to non-hepatic indications [133,134,135,136,137]. Broader independent validation across human-relevant models and future clinical studies will therefore be required to define the therapeutic value of targeting the IL-11/IL-11RA axis in liver disease.

## Figures and Tables

**Table 1:** Evidence supporting protective or pathogenic roles of IL-11 across liver diseases.

Roles in Liver	Liver Disease/Model	Year	Experimental System and Principal Approach	Recombinant IL-11 Exposure: Ligand → Target	Main Finding	Ref.
protective effect	Concanavalin A-induced immune-mediated liver injury and APAP hepatotoxicity	1999; 2001	Mouse models; prophylactic pharmacological administration	rhIL-11 → mouse †	Reduced inflammatory injury, hepatocyte damage, and/or mortality	[40,41]
protective effect	Endotoxin-associated acute liver injury	2004	Rat endotoxemia model; pharmacological administration	rhIL-11 → rat †	Reduced hepatic inflammation and injury	[42]
protective effect, with human association	Chronic HCV infection with advanced liver disease	2004	Open-label study; 20 patients received rhIL-11 for 12 weeks	rhIL-11 → human §	Histological activity and ALT improved in some patients; uncontrolled design and universal lower-extremity edema limit interpretation	[46]
protective effect	Oxidative and APAP-associated liver injury	2012	Mouse injury models; Il11ra1 deficiency and administration of the human IL-11 superagonist NT-3N	Human IL-11-derived NT-3N → mouse †	Linked oxidative-stress-induced IL-11 to STAT3 activation and compensatory hepatocyte proliferation	[45]
protective effect	Hepatic ischemia–reperfusion injury	2015; 2016	Two mouse warm-ischemia/reperfusion studies; mouse hepatocyte experiments	rhIL-11 → mouse/mouse hepatocytes †	Reduced biochemical and histological injury, largely associated with STAT3 activation	[43,44]
indirect protective effect	APAP-induced acute liver injury	2021	Mouse model treated with n-3 polyunsaturated fatty acids; pathway analysis without IL-11-specific rescue	None ‡	Suppression of ERK–Fra-1-dependent IL-11 production and STAT3/Bcl-2 signaling was associated with more severe injury; pleiotropic effects prevent attribution specifically to IL-11	[52]
pathogenic effect	APAP-induced acute liver injury	2021; 2022	Mouse and human hepatocytes; direct cross-species ligand comparison; Il11, hepatocyte-specific Il11ra1 or gp130 deletion; neutralizing antibodies	rmIL-11 → mouse and mouse hepatocytes §; rhIL-11 → human hepatocytes §;	Species-matched IL-11 induced sustained ERK/JNK–NOX4–ROS signaling, hepatocyte death, and impaired regeneration; rhIL-11 antagonized endogenous mouse IL-11	[8,53]
pathogenic effect	Diet-induced steatosis, metabolic dysfunction-associated steatohepatitis (MASH), and fibrosis	2019; 2021	Multiple dietary mouse models; primary human HSCs, human liver slices and hepatocytes; cell-specific deletion and antibody blockade	rmIL-11 → mouse/mouse cells §; rhIL-11 → human liver cells §	IL-11 promoted hepatocyte lipotoxicity, HSC activation, inflammation, steatosis, and fibrosis; genetic or antibody-mediated inhibition improved disease	[25,28]
model-specific limited effect	choline-deficient, L-amino acid-defined, high-fat diet (CDAHFD)-induced MASH and fibrosis	2024	Il11ra1-deficient mice and CDAHFD feeding	None ‡	Il11 was increased, but disruption of IL-11 signaling produced only limited changes in injury, inflammation, fibrosis, and associated signaling	[54]
pathogenic effect, with human association	Alcohol-related liver disease	2023	Human ALD cohorts, primary human hepatocytes, and ethanol-fed mice; IL-11RA blockade	rhIL-11 → primary human hepatocytes §; mouse intervention used IL-11RA blockade rather than exogenous IL-11 ‡	IL-11 correlated with human disease severity and poor transplant-free survival; receptor blockade reduced liver injury, steatosis, and inflammation in mice	[55]
pathogenic effect	Experimental hepatic fibrosis	2023	LX-2-to-LO-2 conditioned-medium system; IL11 knockdown, IL-11 neutralization, and IL11RA knockdown	None ‡	HSC-derived IL-11 promoted hepatocyte apoptosis through ERK/JNK-associated signaling	[48]
pathogenic effect	*S. japonicum*- and CCl_4_-induced fibrosis	2025	Human cirrhotic tissue, two mouse fibrosis models, hepatocyte-specific MCM7 manipulation, and HSC activation assays	rhIL-11 → mouse †	Hepatocyte MCM7 induced IL11 through the SHCBP1–RACGAP1–STAT3 axis; IL-11 neutralization or cross-species antagonism reduced HSC activation and fibrosis	[56]
pathogenic effect	CCl_4_-induced liver fibrosis	2026	AAV6-mediated HSC-directed Il11 overexpression, macrophage depletion, F12 nanobody, LX-2 cells and mouse BMDMs	ligand species not stated	HSC-derived IL-11 promoted HSC activation through gp130–SFK–YAP and amplified profibrotic macrophage responses; F12 reduced fibrosis	[57]
pathogenic effect	Established HCC, postoperative prognosis, and metastasis	2011–2018; 2023	Human tumor cohorts and HCC cell lines; IL11 knockdown, miRNA/lncRNA regulation, and restoration experiments	None ‡	High IL-11 was associated with adverse survival and bone metastasis; tumor-cell-autocrine IL-11–STAT3 signaling promoted survival, proliferation, migration, and metastatic colonization	[49,50,58,59,60,61,62,63,64]
pathogenic effect	Post-hepatectomy HCC recurrence	2019	Hepa1-6 cells and mouse postoperative-recurrence models; Il11ra silencing, neutralization, and STAT3 inhibition	rmIL-11 → mouse Hepa1-6 cells/mice §	IL-11–IL-11RA–STAT3 signaling promoted tumor-cell survival and postsurgical tumor outgrowth; pathway inhibition reduced recurrence	[65,66]
pathogenic effect	Experimental HCC and response to programmed cell death protein 1 (PD-1) blockade	2025	Syngeneic mouse tumor models; anti-IL-11 antibody 9MW3811 alone or with anti-PD-1	None ‡	IL-11 blockade increased cytotoxic T-cell activity and enhanced anti-PD-1 efficacy	[67]

† Species-mismatched exposure: human recombinant IL-11 or a human IL-11-derived agonist was administered to rodent animals or rodent cells. § Species-matched exposure. ‡ No recombinant IL-11 was used as the decisive disease-model perturbation; conclusions were derived from endogenous expression, genetic manipulation, antibody/nanobody blockade, conditioned medium, or clinical association.

**Table 2:** Liver disease-specific IL-11 signaling pathways and their functional consequences.

Pathway/Module	Principal Cell or Model	Liver Disease Context	Mechanism	Functional Consequence	Ref.
JAK–STAT3	Injured and surviving hepatocytes; macrophages; HCC cells; residual tumor cells after hepatectomy	Acute liver injury; liver fibrosis; established and metastatic HCC; postoperative recurrence	IL-11–IL-11Rα–gp130 activates STAT3. In fibrotic liver, MCM7–SHCBP1–RACGAP1 promotes STAT3-dependent IL11 transcription. In HCC cells, autocrine IL-11 sustains STAT3 activation.	Associated with compensatory hepatocyte proliferation; promotes macrophage chemokine production, HCC-cell growth and survival, metastatic colonization, and postoperative recurrence.	[45,56,57,58,59,65,66]
RAS–RAF–MEK–ERK	Hepatocytes; primary human HSCs; human precision-cut liver slices	APAP injury; steatohepatitis; alcohol-related liver disease; liver fibrosis	Hepatocyte-autocrine IL-11 induces sustained ERK signaling. In HSCs, fibrogenic stimuli induce IL-11, which activates ERK and further reinforces IL-11 production.	Amplifies hepatocyte stress and death, restricts regenerative cell-cycle re-entry, and maintains HSC activation, ACTA2 expression, collagen secretion, and matrix invasion. Direct IL-11-dependent ERK signaling in HCC cells remains unestablished.	[8,25,28,53,55,100]
PI3K–AKT–mTOR	Macrophages; liver tissue from experimental ALD models	Liver fibrosis; alcohol-related liver disease	HSC-derived IL-11 promotes macrophage PI3K–mTOR–p70S6K–S6RP signaling and TGF-β synthesis. In experimental ALD, IL-11 suppression occurred concurrently with PI3K–AKT–mTOR activation †.	Promotes profibrotic macrophage polarization, secondary HSC activation, and fibrotic amplification. A direct role in hepatocyte injury or HCC has not been established.	[57,101]
NOX4–ROS–JNK	Mouse and human hepatocytes; lipid-loaded hepatocytes	APAP injury; steatohepatitis; alcohol-related liver disease	IL-11 induces NOX4-derived ROS and ERK/JNK activation, followed by mitochondrial dysfunction, caspase-3 activation, and reduced fatty-acid oxidation.	Promotes hepatocyte death, lipid accumulation, steatosis, inflammation, and persistence of maladaptive repair.	[8,25,28,55]
gp130–SFK–YAP	Primary HSCs; HSC-directed IL-11 overexpression and CCl_4_ fibrosis models	HSC activation; matrix remodeling; liver fibrosis	IL-11 activates an HSC-intrinsic gp130–SFK–YAP pathway. Matrix stiffness may further increase IL11 expression and reinforce YAP-dependent mechanotransduction †.	Promotes HSC contraction, migration, mechanosensitive transcription, collagen synthesis, and persistence of the fibrogenic phenotype.	[25,57]
IL-11R-dependent immune remodeling	HSCs; macrophages; neutrophils; intratumoral CD8^+^ T cells	Steatohepatitis; alcohol-related liver disease; fibrosis; HCC	IL-11 induces HSC CCL2 production and macrophage profibrotic programs. IL-11R blockade reduces inflammatory-cell recruitment and increases cytotoxic CD8^+^ T-cell responses †.	Reinforces stromal–immune crosstalk and fibrosis; IL-11 blockade reduces hepatic inflammation and enhances the antitumor efficacy of PD-1 inhibition.	[25,55,57,67]

† Systemic IL-11R blockade demonstrates immune remodeling but does not identify the principal IL-11 source, the relevant IL-11-responsive cell population, or the intracellular signaling branch responsible for the HCC immune phenotype.

**Table 3:** Current IL-11/IL-11RA-targeted therapeutic strategies.

Strategy Category	Representative Agent/Platform	Molecular Target and Mechanism	Development Stage *	Available Evidence	Main Translational Limitations	Ref.
Neutralizing/blocking antibodies	X203/X209	X203 neutralizes IL-11; X209 blocks IL-11RA, preventing productive receptor signaling	Preclinical	Therapeutic activity in mouse models of APAP injury, MASH, liver fibrosis and ALD; complementary studies in primary human hepatocytes, HSCs and liver slices	No good laboratory practice (GLP) toxicology or clinical liver-disease data; systemic safety, pharmacokinetics and optimal treatment window remain undefined	[8,25,55]
	MAB218	Neutralizing anti-IL-11 antibody	Preclinical	Reduced NET formation and hepatic metastatic burden in a mouse model of colorectal-cancer liver metastasis	Evidence is confined to one metastatic model; activity in primary HCC and human liver metastasis remains untested	[47]
	9MW3811	Neutralizing anti-IL-11 antibody	Phase II, pathological scars	Preclinical antitumor activity with PD-1 blockade in a Hepa1-6 HCC model; Phase I study completed in healthy participants	No clinical efficacy data in HCC or chronic liver disease; optimal combination regimen and responsive cell population remain uncertain	[67,136,137]
	BI 765423	Neutralizing anti-IL-11 antibody	Phase IIa, IPF	Phase I evaluation in healthy participants followed by clinical development in IPF	No published therapeutic evidence in liver disease; hepatic target engagement and long-term systemic safety remain unknown	[133,134]
	LASN01	Blocking anti-IL-11RA antibody	Phase II completed, TED	Phase I target-engagement and safety data; Phase II evidence of clinical-activity-score improvement in TED	Clinical activity has not been tested in liver disease; effects on liver repair, regeneration and host defense require evaluation	[135]
Nucleic acid-based suppression of IL-11 signaling	siIl11@NP-AEAA/siIl11ra1@NP-AEAA	Activated-HSC-targeted delivery of siRNAs against Il11 or Il11ra1	Preclinical proof-of-concept	Reduced IL-11–ERK signaling, HSC activation and fibrosis in two mouse MASH models	Delivery in advanced cirrhotic liver, endosomal escape, off-target silencing, innate immune activation and scalable manufacturing require validation	[118]
	mIL11-scFv@AA3G	Liver-directed mRNA delivery encoding a neutralizing anti-IL-11 scFv	Preclinical proof-of-concept	Sustained intrahepatic scFv production and therapeutic activity in early and fibrotic mouse MASH models	Control of expression level and duration, repeat dosing, vehicle or product immunogenicity and applicability to human cirrhosis remain unresolved	[119]
Engineered IL-11 antagonists and peptide-based inhibitors	IL-11Δ10/Mutein; engineered IL-11 decoy; W147C dimer; IL-11–6M/analogue 13	Engineered IL-11 variants retain receptor engagement while disrupting productive gp130 signaling-complex assembly or reducing gp130 recruitment	Preclinical	Structural and cellular validation; therapeutic proof of concept in lung adenocarcinoma, colorectal cancer, pulmonary fibrosis and renal fibrosis	Residual agonism must be excluded; pharmacokinetics, immunogenicity and tissue exposure require optimization; no direct therapeutic validation in liver disease	[17,112,120,121,122]
	Cyclic peptides 4/15; 4L2/4L2-P13D; P19	Peptide-based disruption of IL-11–IL-11RA interaction or IL-11 signaling	Discovery to preclinical	Peptides 4/15 established biochemical inhibition; later macrocyclic and IL-11-derived peptides showed cellular antagonism and antifibrotic efficacy in renal fibrosis models	Proteolytic stability, pharmacokinetics, selectivity, delivery and long-term safety remain uncertain; no liver-disease or clinical validation	[123,124,125]
IL-11RA-directed targeting and payload delivery	BMTP-11	IL-11RA-binding peptidomimetic that delivers a pro-apoptotic payload to receptor-expressing cells	Phase 0 completed	Target localization and apoptosis assessed in six patients with metastatic castration-resistant prostate cancer	Antitumor efficacy was not established; reversible dose-limiting nephrotoxicity and possible on-target toxicity in IL-11RA-expressing hepatocytes and HSCs	[126,127]
	IL-11RA CAR-T cells	CAR-T cells recognize and eliminate IL-11RA-expressing tumor cells	Preclinical	Antitumor activity in osteosarcoma and experimental lung-metastasis models	No validation in HCC; antigen heterogeneity, immunosuppressive liver microenvironment and on-target/off-tissue hepatic toxicity are major concerns	[128]
	^153^Sm-DTPA-c(CGRRAGGSC)	IL-11RA-binding cyclic peptide delivers a therapeutic radionuclide to receptor-expressing tumor cells	Preclinical	Selective tumor accumulation and growth inhibition in an MHCC97-H liver-cancer xenograft model	Evidence is limited to xenografts; efficacy and safety in orthotopic, immunocompetent or fibrosis-associated HCC remain unknown	[129]
	IL11-PDox and related IL-11/IL-11RA-guided nanocarriers	Receptor-guided delivery of chemotherapeutic payloads through polymersomes, engineered membrane-coated nanoparticles or liposomal systems	Preclinical	Antitumor activity demonstrated predominantly in osteosarcoma models, including recurrent, metastatic and patient-derived tumors	Limited evidence outside selected IL-11RA-high tumors; biodistribution, receptor heterogeneity and on-target, off-tumor toxicity require evaluation	[130,131,132]

* Development stages are reported as of August 2026.

## Data Availability

No new data were created or analyzed in this study.
